# Impaired protein hydroxylase activity causes replication stress and developmental abnormalities in humans

**DOI:** 10.1172/JCI152784

**Published:** 2023-04-03

**Authors:** Sally C. Fletcher, Charlotte Hall, Tristan J. Kennedy, Sander Pajusalu, Monica H. Wojcik, Uncaar Boora, Chan Li, Kaisa Teele Oja, Eline Hendrix, Christian A.E. Westrip, Regina Andrijes, Sonia K. Piasecka, Mansi Singh, Mohammed E. El-Asrag, Anetta Ptasinska, Vallo Tillmann, Martin R. Higgs, Deanna A. Carere, Andrew D. Beggs, John Pappas, Rachel Rabin, Stephen J. Smerdon, Grant S. Stewart, Katrin Õunap, Mathew L. Coleman

**Affiliations:** 1 Institute of Cancer and Genomic Sciences, University of Birmingham, Birmingham, United Kingdom.; 2 Department of Clinical Genetics, Genetics and Personalized Medicine Clinic, Tartu University Hospital, Tartu, Estonia.; 3 Institute of Clinical Medicine, University of Tartu, Tartu, Estonia.; 4 Center for Mendelian Genomics, Broad Institute of MIT and Harvard, Cambridge, Massachusetts, USA.; 5 Divisions of Newborn Medicine and Genetics and Genomics, Department of Pediatrics, Boston Children’s Hospital and Harvard Medical School, Boston, Massachusetts, USA.; 6 Faculty of Science, Benha University, Benha, Egypt.; 7 Children’s Clinic, Tartu University Hospital, Tartu, Estonia.; 8 GeneDx, Gaithersburg, Maryland, USA.; 9 Clinical Genetic Services, Department of Pediatrics, NYU Langone Medical Center, New York, New York, USA.

**Keywords:** Cell Biology, Genetics, DNA repair, Genetic diseases, Genetic instability

## Abstract

Although protein hydroxylation is a relatively poorly characterized posttranslational modification, it has received significant recent attention following seminal work uncovering its role in oxygen sensing and hypoxia biology. Although the fundamental importance of protein hydroxylases in biology is becoming clear, the biochemical targets and cellular functions often remain enigmatic. JMJD5 is a “JmjC-only” protein hydroxylase that is essential for murine embryonic development and viability. However, no germline variants in JmjC-only hydroxylases, including *JMJD5*, have yet been described that are associated with any human pathology. Here we demonstrate that biallelic germline *JMJD5* pathogenic variants are deleterious to JMJD5 mRNA splicing, protein stability, and hydroxylase activity, resulting in a human developmental disorder characterized by severe failure to thrive, intellectual disability, and facial dysmorphism. We show that the underlying cellular phenotype is associated with increased DNA replication stress and that this is critically dependent on the protein hydroxylase activity of JMJD5. This work contributes to our growing understanding of the role and importance of protein hydroxylases in human development and disease.

## Introduction

The enzymatic incorporation of a single oxygen atom into an amino acid side chain is a relatively poorly characterized but emerging posttranslational modification (PTM) (1). Seminal work on the role of protein hydroxylation in oxygen sensing and the hypoxia response has highlighted the importance of this enigmatic PTM in physiology and disease (2, 3).

Protein hydroxylation is generally catalyzed by members of the 2-oxoglutarate–dependent (2OG-dependent) oxygenase family. These enzymes require fundamental nutrients and metabolites for activity, including molecular oxygen, Fe(II), and the Krebs cycle intermediate 2OG (4, 5). Human 2OG-oxygenases catalyze a variety of oxidative modifications in proteins, nucleic acids, and lipids that generally result in either stable hydroxyl modifications or unstable hydroxymethyl intermediates (4, 6). The latter form the basis of demethylation reactions that represent a critical component of a core regulatory network controlling epigenetics (7).

2OG-oxygenases that catalyze histone lysine demethylation (KDMs) belong to a subfamily known as Jumonji-C (JmjC) enzymes (7). They are so called because the characteristic double-stranded β-helix fold of their catalytic domains resembles that of the founding enzyme, Jumonji (JARID2). Consistent with important roles in epigenetic regulation, KDMs are often essential for life and are widely implicated in human development and disease (8, 9). As such, KDMs have been comprehensively studied, unlike other subtypes of 2OG-oxygenases.

There is one other subfamily of 2OG-oxygenases that share homology with KDMs in their catalytic domains. This class lack functional domains commonly identified in KDMs and so were originally named “JmjC-only” enzymes (7). These 2OG-oxygenases remain relatively poorly characterized compared with KDMs, with some members still considered as “orphans” (6, 10, 11). Recent structural and biochemical studies indicate that they catalyze stable hydroxylation and possess important functional domains in addition to the JmjC catalytic domain (12–18). Interestingly, the structural studies also suggest that KDMs evolved from the JmjC-only hydroxylase family (12). Whether this evolutionary relationship is consistent with JmjC-only hydroxylases being as fundamentally important as KDMs is not yet clear, though emerging evidence is consistent with critical roles for JmjC-only hydroxylases in a variety of essential cellular processes (1, 6, 10, 11). However, mutation of a JmjC-only hydroxylase has not yet been identified as the basis of a Mendelian human disease.

*JMJD5* is a highly conserved gene (19, 20) and is the only member of the JmjC-only hydroxylase family that has been demonstrated to be essential for early mouse embryonic development. Homozygous *Jmjd5* gene knockout causes severe growth retardation during embryogenesis and lethality in mid-gestation (21, 22), which has been linked to increased genome instability and activation of the p53/p21 pathway (19, 23, 24). Although the molecular mechanisms involved remain unclear, detailed structural and biochemical studies suggest that JMJD5 is a protein hydroxylase (13, 17, 18). A recent study confirmed this protein hydroxylase assignment and demonstrated that JMJD5 possesses arginyl hydroxylase activity (18), which thus far is unique in humans. Nonetheless, whether this hydroxylase activity is required for the essential roles of JMJD5 in mouse embryonic development is not yet known.

Here, we describe JMJD5 as the first member of the JmjC-only hydroxylase family of 2OG-oxygenases, to our knowledge, to be the basis of a human developmental disorder. We identify biallelic variants in patients with a variety of abnormalities, including intrauterine and perinatal growth delay, relative macrocephaly, intellectual disability, and facial dysmorphism. We show that the identified variants severely compromise normal JMJD5 mRNA splicing and protein stability, rendering cells hypomorphic for JMJD5 function. Lastly, we demonstrate that patient-derived cells exhibit increased levels of replication stress and genome instability in a manner that is critically dependent on the hydroxylase activity of JMJD5. Taken together, our work identifies JMJD5 as a critical regulator of the replication stress response that is essential for suppressing neurodevelopmental abnormalities in humans.

## Results

### Identification of biallelic JMJD5 variants in patients with developmental abnormalities.

**Figure 1:**
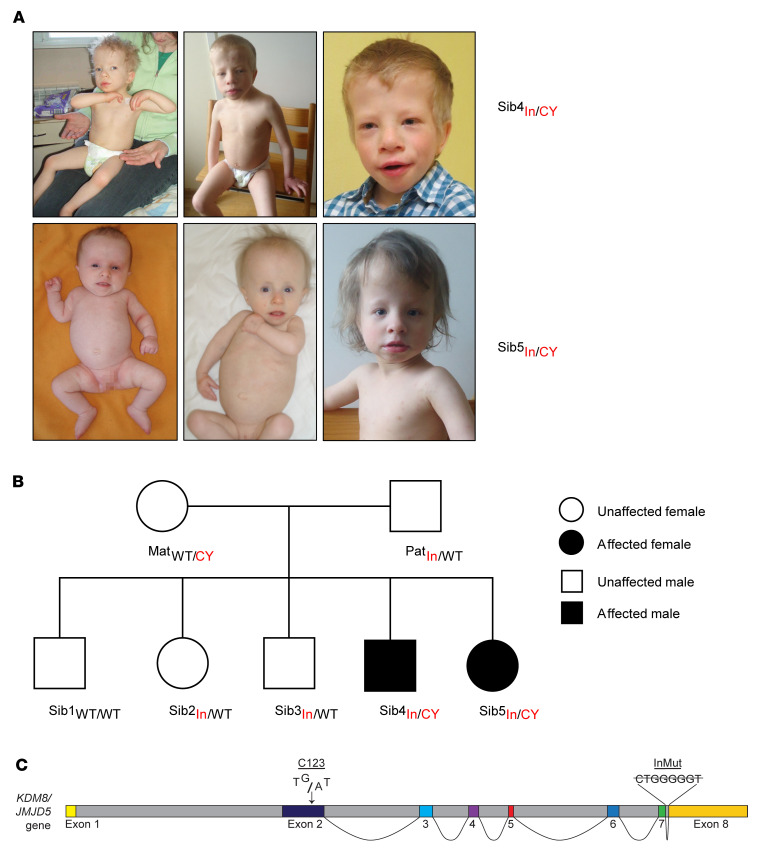
Heritable *JMJD5* pathogenic variants are carried by 2 patients with a novel developmental disorder. (**A**) Photographs of 2 affected individuals (Sib4_In/CY_ and Sib5_In/CY_) demonstrating common phenotypes including relative macrocephaly and facial dysmorphism (also see Supplemental Figure 1 and Supplemental Patient Case Reports). Top: Affected male Sib4_In/CY_ at age 3 years and 3 months (left) and age 10 years (middle and right). Bottom: Affected female Sib5_In/CY_ at ages 2 months (left), 15 months (middle), and 7 years (right). (**B**) Pedigree of the family. The *JMJD5* genotype is annotated for each individual (In, intronic variant; CY, C123Y variant; Mat, maternal; Pat, paternal; Sib, sibling). (**C**) Graphical representation of the *JMJD5* gene structure and the position of the 2 variants. The C123Y variant is localized within exon 2, which encodes most of the N-terminus (Figure 2). The intronic deletion is located between exons 7 and 8.

Here we present a family that experienced 2 pregnancies associated with intrauterine hypotrophy, oligohydramnios, and growth delay. Postnatal presentation included severe failure to thrive, relative macrocephaly, facial dysmorphism (including a triangular face, high forehead, microretrognathia, short neck, and dysplastic ears), mild brain atrophy, mild to moderate intellectual disability, and muscular hypotonia (Figure 1A and Supplemental Figures 1 and 2; supplemental material available online with this article; https://doi.org/10.1172/JCI152784DS1; also see detailed Supplemental Patient Case Reports). Growth hormone replacement therapy had a positive impact on the growth and hypotonia of both affected siblings (Supplemental Figure 2 and Supplemental Patient Case Reports).

The combined clinical symptoms were consistent with a medical classification of syndromic short stature, with some associated neurodevelopmental anomalies. The clinical presentation also had some similarities to Silver-Russell, Mabry, and Coffin-Siris syndromes (Supplemental Patient Case Reports). Silver-Russell and Coffin-Siris syndromes were ruled out by genomic analyses of molecular defects associated with those conditions (Supplemental Figure 3), including hypomethylation of the 11p15 chromosomal region and UPD7/14 molecular tests, which were both normal. Mabry syndrome was excluded by the absence of hyperphosphatasia.

To identify genetic variants segregating with the disease, we performed whole genome sequencing (WGS) on quadruplicate samples of genomic DNA purified from the 2 affected siblings and both parents. WGS reads were aligned to the reference genome build hg38 before calling and identification of rare variants with a minor-allele frequency less than 0.01. This analysis failed to detect disease-segregating variants in any known genes associated with short stature syndromes, growth hormone signaling, primordial dwarfism, or known components of the DNA replication initiation complex (Supplemental Figure 3 and data not shown). Rather, we discovered biallelic variants in the *JMJD5* gene of both affected patients (c.1086+14_1200_21del and c.482G>A, p.Cys123Tyr) that were inherited from the parents and segregated with the disease (Figure 1B and Supplemental Figures 4 and 5). No other inherited variants were identified that segregated with the disease.

Before this study, homozygous damaging variants had yet to be identified in *JMJD5* (Genome Aggregation Database [gnomAD] v2.1.1 and v3.1). Both of the variants identified here are extremely rare, with allele frequencies of 2.48 × 10^–5^ (c.1086+14_1200_21del, Single Nucleotide Polymorphism Database [dbSNP] rs947596337) and 1.48 × 10^–4^ (c.482G>A, p.Cys123Tyr, dbSNP rs201012033). The *JMJD5* gene has a residual variation intolerance score of –0.6 (i.e., in the top 20% of human genes most intolerant to genetic variation) (genic-intolerance.org) and a predicted loss of function (pLoF) observed/expected score of 0.5 (gnomAD), indicating that the *JMJD5* gene is a target of strong negative selection.

The *JMJD5* gene (NCBI 79831) consists of 8 exons (7 coding) (Figure 1C) on chromosome 16p12.1 that are transcribed into a ubiquitously expressed mRNA (Supplemental Figure 6) (ENST00000286096.8, NM_024773, transcript variant 2). The paternally inherited *JMJD5* variant is a deletion in intron 7–8 (c.1086+14_1200_21del) in close proximity to the boundary with exon 7 (Figure 1C). Since exon/intron boundaries encode important regulatory sequences required for high-fidelity splicing, we assessed the potential functional consequences of the intronic deletion on *JMJD5* pre-mRNA splicing using the Human Splicing Finder (HSF) tool (25). HSF predicted high-scoring donor, acceptor, branchpoint, and enhancer sequences within this region of the exon 7/intron 7–8 boundary (Supplemental Figure 7A). Importantly, HSF also predicted that the intronic variant would ablate a high-scoring enhancer sequence (Supplemental Figure 7, A and B), which would be predicted to affect the specificity and/or efficiency of splicing at proximal splice sites.

**Figure 2:**
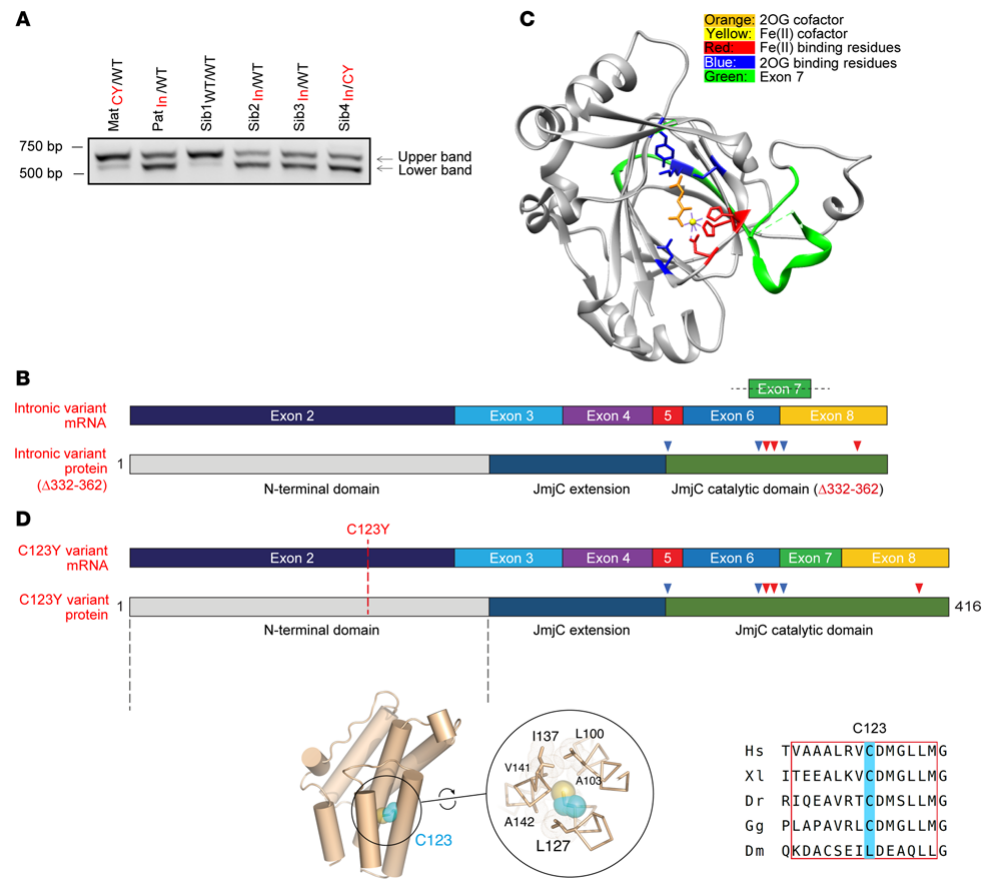
Patient variants affect JMJD5 mRNA splicing and enzyme structure. (**A**) Carriers of the intronic variant express an altered JMJD5 mRNA (lower band). JMJD5 cDNA from exon 3 to the 3′-UTR was amplified from the indicated samples and separated by gel electrophoresis. The coding sequence of the wild-type (WT) JMJD5 mRNA (ENST00000286096.8, NM_024773) spanning this region is 706 bp (upper band). CY, C123Y; In, intronic mutant. (**B**) Graphical representation of the incorrectly spliced intronic variant mRNA (top) and protein (bottom) demonstrating the loss of sequence integral to the catalytic domain (compare with the correctly spliced mRNA in **D**). Fe(II)- and 2OG-binding residues are marked by red and blue arrowheads, respectively. (**C**) Structure of the catalytic domain of JMJD5 (Protein Data Bank: 4GAZ) with critical catalytic residues labeled. The region encoded by exon 7 that is missing from JMJD5^Δ332–362^ is highlighted in green. (**D**) Graphical representation of the C123Y JMJD5 mRNA and protein. The position of the C123Y missense variant within exon 2 and the JMJD5 N-terminus is highlighted (top). Structural predictions suggest that the JMJD5 N-terminus is predominantly α-helical (bottom left; also see Supplemental Figure 12). C123 is highly conserved (bottom right) and predicted to be located within a hydrophobic environment on one side of an amphipathic α-helix within a TPR domain (bottom middle; also see Supplemental Figure 12).

To investigate the impact of the intronic variant on splicing, we studied JMJD5 mRNA in patient-derived cells. To this end we first established and genotyped hTERT-immortalized dermal fibroblasts (Supplemental Figure 8) (we were unable to obtain immortalized fibroblasts from one of the affected patients, Sib5_In/CY_, because of premature senescence). JMJD5 cDNA from exon 3 to the 3′-UTR was then amplified from these cells in an endpoint PCR assay and analyzed by agarose gel electrophoresis. This identified a smaller PCR product that was substantially more abundant in individuals carrying the intronic variant (Figure 2A). Sanger sequencing demonstrated that this mRNA product was identical in all carriers of the intronic variant and was due to the loss of 93 nucleotides encoded predominantly by exon 7 (Supplemental Figure 9), which is consistent with deletion of a critical splicing enhancer sequence in intron 7–8 (Supplemental Figure 7B). We observed the same pattern of alternate splicing in primary cells derived from both affected patients (Supplemental Figure 10A). Unbiased exon usage analysis of RNA sequencing data independently confirmed reduced exon 7 use in cells derived from individuals carrying the intronic variant (Supplemental Figure 10B). Although endpoint PCR assays also detected low levels of this variant in cells derived from patients who did not carry the intronic variants (as confirmed by Sanger sequencing) (Figure 2A and Supplemental Figure 10A), it is likely that this represents a rare transcript in healthy individuals since it is not annotated in gene expression databases.

The in-frame deletion resulting from inappropriate removal of exon 7 deletes amino acids 332 to 362, which reside within a highly conserved (Supplemental Figure 11) and essential section of the JmjC domain that contains a critical catalytic residue (K336) responsible for binding the cofactor 2OG (Figure 2, B and C) (13, 17, 18). We will refer to the protein product of this aberrant splicing event as JMJD5^Δ332–362^. Based on the established importance of the double-stranded β-helix core fold of 2OG-oxygenases (4, 26), we predicted that this deletion is likely to be highly damaging to both the hydroxylase activity and structural stability of JMJD5.

The maternally inherited *JMJD5* variant is a single-nucleotide substitution in exon 2 (c.482G>A, p.Cys123Tyr) (Figure 1C) that causes the substitution of a highly conserved cysteine residue in the N-terminus of the resulting protein product (Q8N371-1) (Supplemental Figure 12A). The resulting missense substitution (C123Y) was predicted by both PolyPhen-2 (Polymorphism Phenotyping v2) and SIFT (Sorting Intolerant from Tolerant) to be highly damaging (scores of 0 and 1, respectively). However, no crystal or NMR structures of any part of the JMJD5 N-terminal region are currently available, precluding direct tertiary structural analysis. Nonetheless, our preliminary sequence homology along with secondary and tertiary structure analyses (Supplemental Figure 12) strongly suggested the presence of a tetratricopeptide (TPR) domain consistent with recently available AlphaFold (27) structural models (Figure 2D). Overall, these observations suggest that C123 is located within the penultimate predicted amphipathic helix, where it is buried within a hydrophobic environment that contributes to the structural core of a TPR-like domain (Figure 2D and Supplemental Figure 12B). Consistent with this, C123 is replaced by small/medium-sized hydrophobic residues in orthologs in the few species (e.g., *Drosophila melanogaster*) where variations at this position are seen (Figure 2D and Supplemental Figure 12A). Replacement with the bulkier tyrosine residue in the C123Y mutant would be expected to be highly structurally disruptive.

### JMJD5 patient variants reduce protein stability and hydroxylase activity.

**Figure 3:**
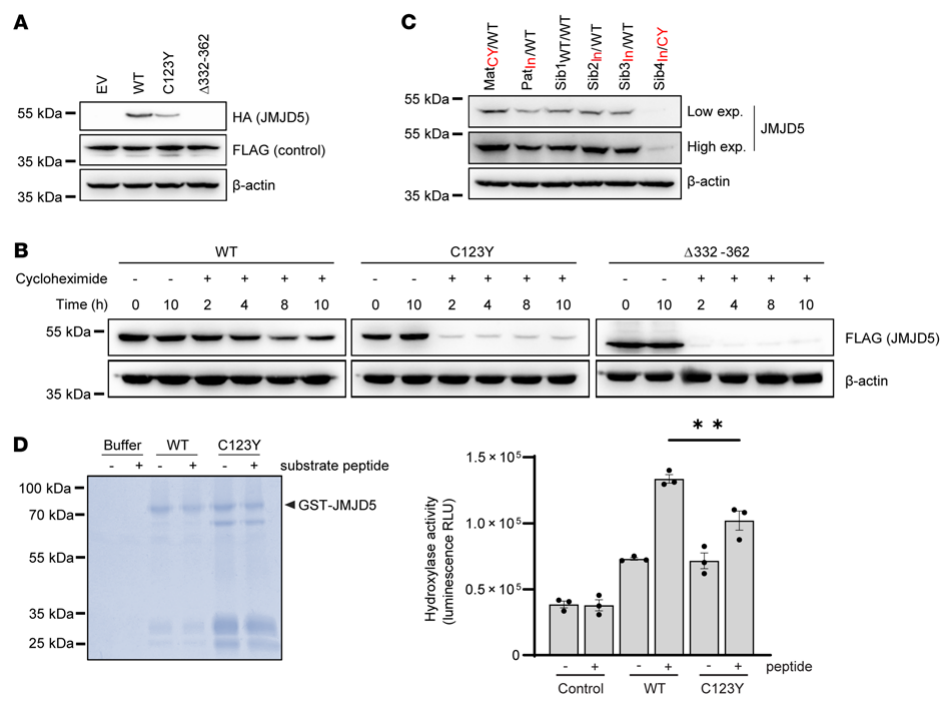
Patient pathogenic variants reduce JMJD5 protein stability and activity. (**A**) C123Y and JMJD5^Δ332–362^ variants reduce JMJD5 expression. Western blotting following transfection of HA-tagged JMJD5 cDNAs and a transfection control (FLAG-JMJD7) into HeLa cells. (**B**) C123Y and JMJD5^Δ332–362^ are more rapidly degraded. Doxycycline-inducible FLAG-tagged WT, C123Y, or JMJD5^Δ332–362^ cDNAs were introduced into Sib1_WT/WT_ fibroblasts. Cells were treated with 1 μg/mL doxycycline for 16 hours and then 50 μg/mL cycloheximide, to inhibit protein synthesis. Because the C123Y and JMJD5^Δ332–362^ proteins were expressed markedly less than WT (consistent with **A**), longer exposures are included for the C123Y and JMJD5^Δ332–362^ insets (top middle and top right) to support a more direct comparison of the three JMJD5 species. (**C**) Reduced JMJD5 expression in fibroblasts derived from an affected patient. Western blotting for endogenous JMJD5 in immortalized fibroblast cell lines. “Low” and “high” refer to the exposure length. Also see Supplemental Figure 20A. (**D**) Partially purified recombinant JMJD5 C123Y has reduced hydroxylase activity in vitro. GST-tagged WT and C123Y JMJD5 were expressed in *E*. *coli* and purified before in vitro activity assays. Each reaction was also analyzed by Coomassie gel (left). Note the presence of a smaller product (~65 kDa) in the C123Y sample. This may be a cleavage product or chaperone contamination, perhaps indicative of improper folding. The amount of recombinant JMJD5 sample added to the activity assay and Coomassie gel was equalized. Activity was monitored using the Succinate-Glo assay, which measures succinate production (right). Data represent mean ± SEM from 3 independent experiments. Statistical analysis used 1-way ANOVA with Tukey’s post hoc test, with *P* ≤ 0.05 considered significant; **P ≤ 0.01.

To explore the functional effects of the intronic and C123Y variants on JMJD5 protein function, we first cloned the corresponding cDNAs into HA-tagged expression vectors. Consistent with the dramatic changes in protein sequence and JmjC fold caused by the intronic variant, JMJD5^Δ332–362^ was very poorly expressed (Figure 3A and Supplemental Figure 13, A and B), owing to reduced stability (Figure 3B) and increased proteasomal degradation (Supplemental Figure 13C). Interestingly, the C123Y variant also reduced JMJD5 protein levels (Figure 3A and Supplemental Figure 13A) and stability (Figure 3B), although largely in a proteasome-independent manner (Supplemental Figure 13C). Together, these results suggest that at least one important consequence of the biallelic *JMJD5* pathogenic variants observed in these patients is likely to be reduced protein stability. Therefore, we measured endogenous JMJD5 protein levels in the immortalized patient-derived dermal fibroblasts described above. Consistent with this hypothesis, JMJD5 protein levels were substantially lower in cells derived from the affected patient (Sib4_In/CY_) compared with those from either wild-type individuals or heterozygous carriers of the C123Y or intronic variants (Figure 3C). This reduction occurred without noticeable decrease in mRNA expression (Supplemental Figure 14). Whether the residual JMJD5 protein present in cells from the affected patient represents the missense and/or deletion variant is unclear. However, the expressed JMJD5 protein appears to migrate at a similar molecular weight to that observed in wild-type cells (Figure 3C), which may be more consistent with a missense substitution than a deletion variant. Furthermore, the C123Y variant appears to be more stable than the intronic variant (Figure 3A and Supplemental Figure 13). In circumstances when we were able to detect any residual HA-JMJD5^Δ332–362^ expression, it was noticeably smaller in size than the wild-type protein. Together, this may suggest that the C123Y variant is more likely to contribute to residual endogenous protein expression in the affected patient-derived cells than the JMJD5^Δ332–362^ variant. Therefore, we next sought to determine whether the C123Y variant influences other aspects of JMJD5 function.

Since the N-terminus of JMJD5 is reported to contain both nuclear localization and export sequences (28), we tested whether the C123Y variant affected the subcellular distribution of JMJD5. However, the predominantly nuclear localization of JMJD5 was not altered by the C123Y variant (Supplemental Figure 15). The JMJD5 N-terminus has also been implicated in binding RCC1 domain–containing protein 1 (RCCD1) (29), but this interaction was likewise unaffected by this variant (Supplemental Figure 16).

Since regions outside the catalytic domain of 2OG-oxygenases can contribute to substrate binding and catalysis (26), we explored whether the C123Y variant had any impact on JMJD5 hydroxylase activity. We first purified recombinant wild-type and C123Y mutant JMJD5 from *E*. *coli* and performed in vitro activity assays against a synthetic peptide substrate (18). Interestingly, we observed a second major protein species in purified samples of the JMJD5 C123Y protein (Figure 3D, left panel), and a substantially reduced purification yield, perhaps consistent with inappropriate folding and the reduced stability observed in cells. In this context, we observed a modest but statistically significant reduction in hydroxylase activity of the C123Y mutant (Figure 3D, right panel). Attempts to purify and assay the activity of the recombinant JMJD5^Δ332–362^ protein were unsuccessful owing to very poor yield and degradation, which may suggest inherent structural instability of this variant.

Taken together, the structural, bioinformatic, and biochemical analyses described above suggest that both *JMJD5* patient variants substantially damage the normal expression and hydroxylase activity of JMJD5 protein, consistent with their being the genetic basis of the disease presented. To understand how these hypomorphic JMJD5 variants might drive the clinical phenotype, we next sought to investigate the cellular consequences of their expression.

### Reduced JMJD5 expression is associated with increased replication stress.

**Figure 4:**
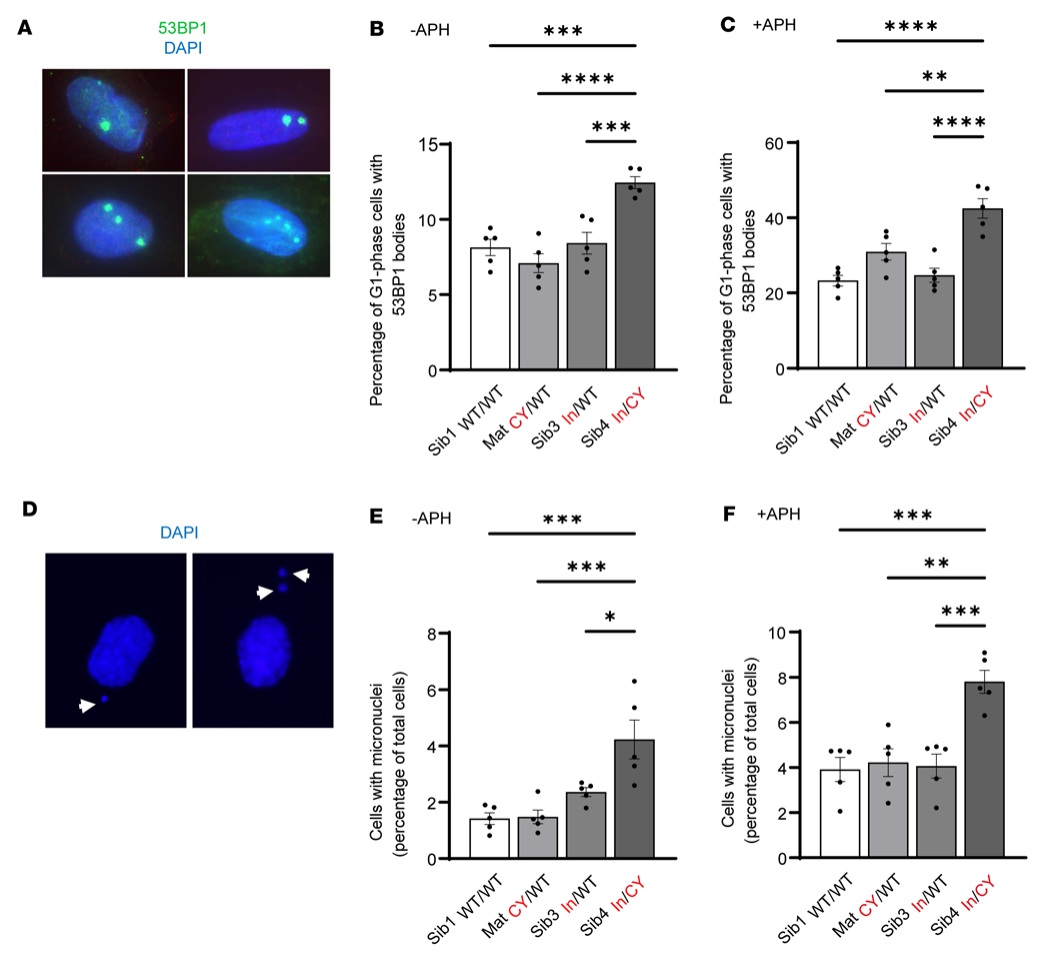
Biallelic *JMJD5* pathogenic variants are associated with increased markers of replication stress. Immortalized fibroblasts were monitored for 2 commonly used markers of replication stress (micronuclei and 53BP1 bodies). The affected Sib4_In/CY_ fibroblasts show significantly increased replication stress. (**A**) 53BP1 bodies were counted using immunofluorescence staining and distinguished from 53BP1 foci based on their size and presence only in G_1_ cells (using costaining for CENPF). Shown are examples of cells with different numbers of 53BP1 bodies. (**B**) 53BP1 bodies in untreated cells were significantly increased in Sib4_In/CY_ immortalized fibroblasts. APH indicates aphidicolin, a DNA polymerase inhibitor that is an established replication stress stimulus (see below). (**C**) 53BP1 bodies were also significantly increased in Sib4_In/CY_ immortalized fibroblasts treated with APH (0.5 μM for 48 hours). (**D**) Micronuclei were counted following DAPI staining. Shown are 2 examples of cells with micronuclei. (**E**) Micronuclei were significantly increased in untreated Sib4_In/CY_ immortalized fibroblasts. (**F**) Micronuclei were also significantly increased in Sib4_In/CY_ immortalized fibroblasts treated with APH. (**B**, **C**, **E**, and **F**) Data represent mean ± SEM from 5 independent experiments. For 53BP1 bodies, a minimum of 300 cells were counted per sample. For micronuclei, a minimum of 500 cells were counted per sample. Statistical analyses used 1-way ANOVA with Tukey’s post hoc test; *P ≤ 0.05, **P ≤ 0.01, ***P ≤ 0.001, ****P ≤ 0.0001.

Given that JMJD5 has been implicated in DNA damage repair and maintenance of genome stability (19, 23, 24), we explored whether DNA damage and/or genome instability were associated with the *JMJD5* variants in our patient-derived cell models. Interestingly, we observed that the immortalized cell line derived from one of the affected individuals exhibited increased levels of spontaneous G_1_-phase p53-binding protein (53BP1) “bodies” (Figure 4, A and B), which are commonly used as a marker of unresolved replication stress (RS) (defined as any perturbation that slows or stalls DNA replication) (30). Moreover, the increase in formation of the spontaneous 53BP1 bodies observed in cells from the affected patient was dramatically elevated following exposure to the DNA polymerase inhibitor aphidicolin, an established exogenous RS stimulus (Figure 4C). Notably, the RS phenotype in these cells was not associated with an overt proliferative defect or cell cycle disturbance (Supplemental Figure 17). Together, these results suggest that the affected patient cells exhibit signs of increased cellular RS.

**Figure 5:**
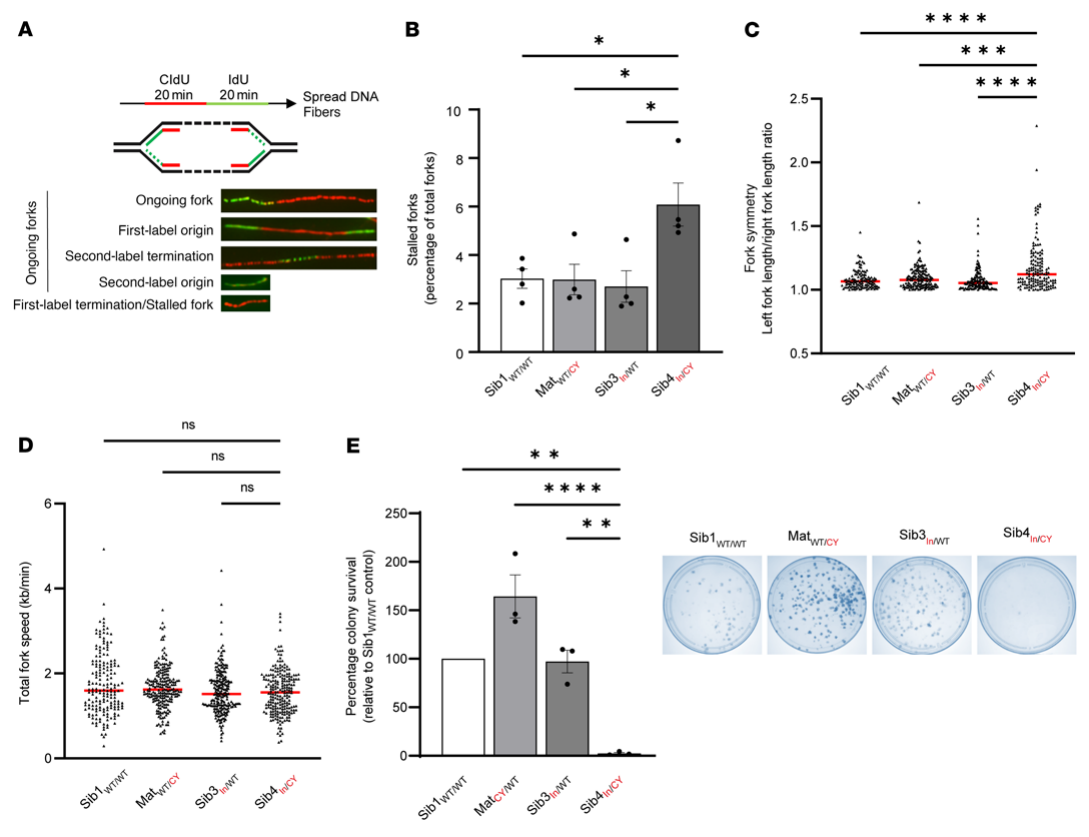
Biallelic *JMJD5* pathogenic variants are associated with impaired DNA replication fidelity and reduced colony survival. (**A**) DNA fiber assays were performed by incubation of cells with thymidine analog CldU followed by IdU, with detection by immunofluorescence (top). Examples of different labeling outcomes are shown (bottom). (**B**) Stalled replication forks were significantly increased in Sib4_In/CY_ immortalized fibroblasts. The prevalence of stalled forks was calculated as a percentage of total fork structures counted. A minimum of 200 forks were counted per condition in each experiment. (**C**) Asymmetric replication forks were significantly increased in Sib4_In/CY_ immortalized fibroblasts. Fork symmetry was determined by measurement of the ratio between the two IdU tracts found in first-label origin structures. Symmetric forks should approach a ratio of about 1.0. An increase indicates fork asymmetry. At least 50 structures were measured per sample in each experiment. (**D**) The total length of ongoing DNA fiber fork structures was measured and converted to replication fork speed. No significant difference in replication fork speed was observed. A minimum of 200 forks were analyzed per sample. (**E**) Fibroblasts were plated at limited density and monitored for colony formation. Sib4_In/CY_ fibroblasts had reduced colony formation. (**B**–**D**) Data represent mean ± SEM from 4 independent experiments. (**E**) Data represent mean ± SEM from 3 independent experiments. Statistical analyses used 1-way ANOVA with Tukey’s post hoc test (**B** and **E**) or Kruskal-Wallis with Dunn’s correction (**C** and **D**); *P ≤ 0.05, **P ≤ 0.01, ***P ≤ 0.001, ****P ≤ 0.0001.

Unresolved RS can lead to genome instability via DNA breakage and formation of extranuclear DNA bodies known as micronuclei (31). Consistent with elevated levels of RS, we also observed a significant increase in the basal and aphidicolin-induced frequency of micronuclei in cells derived from the affected patient (Figure 4, D–F). Since micronuclei can arise from either mitotic defects or unrepaired DNA damage (31), we sought to define the origin of the increased spontaneous micronuclei observed in immortalized cells derived from the affected patient. To achieve this, we stained cells with antibodies against CENPA (which marks micronuclei arising from missegregated chromosomes retaining a centromere) and 53BP1 (which marks micronuclei originating from damaged chromosomes containing a double-strand break) (32). Importantly, we observed a significant increase in the frequency of spontaneous 53BP1-positive CENPA-negative micronuclei in immortalized cells derived from the affected patient (Supplemental Figure 18). These observations are consistent with the micronuclei arising as a consequence of unrepaired DNA damage and are more indicative of a direct role for JMJD5 in maintaining the fidelity of DNA replication. Consistent with this hypothesis, single-molecule DNA fiber analysis (Figure 5A) demonstrated a significant increase in the level of spontaneously stalled replication forks in cells derived from the affected patient (Figure 5B), which was exacerbated following treatment with the ribonucleotide reductase inhibitor hydroxyurea (Supplemental Figure 19A).

Normal DNA replication proceeds bidirectionally and with comparable speeds from the same origin, resulting in fork “symmetry.” Reduced replication fork stability can present as replication fork asymmetry due to slowing, pausing, or collapse of forks on one side of an origin. Consistent with increased replication fork instability, we observed significantly increased spontaneous and hydroxyurea-induced fork asymmetry in cells derived from the affected patient (Figure 5C and Supplemental Figure 19B), despite the overall DNA replication fork speed being unaffected (Figure 5D and Supplemental Figure 19C). However, we note that global fork speed can appear unchanged during RS, despite a detectable increase in fork stalling and asymmetry (33). To explore whether the increased RS associated with the hypomorphic *JMJD5* variants might have an impact on cell viability, we performed colony survival assays. We observed a correlation between reduced JMJD5 expression and decreased colony survival of patient-derived fibroblasts (Figure 5E).

Overall, these data suggest that compromised *JMJD5* function is associated with increased RS in immortalized fibroblasts derived from the affected patient Sib4_In/CY_. Importantly, this phenotype was not restricted to immortalized cells: primary fibroblasts derived from either affected patient (Sib4_In/CY_ and Sib5_In/CY_) also expressed reduced JMJD5 protein levels (Supplemental Figure 20A) and displayed increased basal and aphidicolin-induced micronuclei (Supplemental Figure 20B). As in immortalized affected cells, the increased micronuclei in primary affected cells were associated with an increase in 53BP1-positive CENPA-negative micronuclei (Supplemental Figure 20C), consistent with RS. Likewise, we observed increased spontaneous 53BP1 bodies (Supplemental Figure 20D) and replication fork asymmetry and stalling in these cells (Supplemental Figure 20, E and F). Importantly, we could recapitulate the RS phenotype associated with JMJD5 dysfunction observed in patient cells in an independent cell type using siRNA-mediated depletion of JMJD5 (Supplemental Figure 21).

Taken together, these data suggest that increased RS is associated with impaired JMJD5 function in a variety of cell types.

### Increased replication stress and reduced cell viability in affected patient cells are dependent on loss of JMJD5 hydroxylase activity.

**Figure 6:**
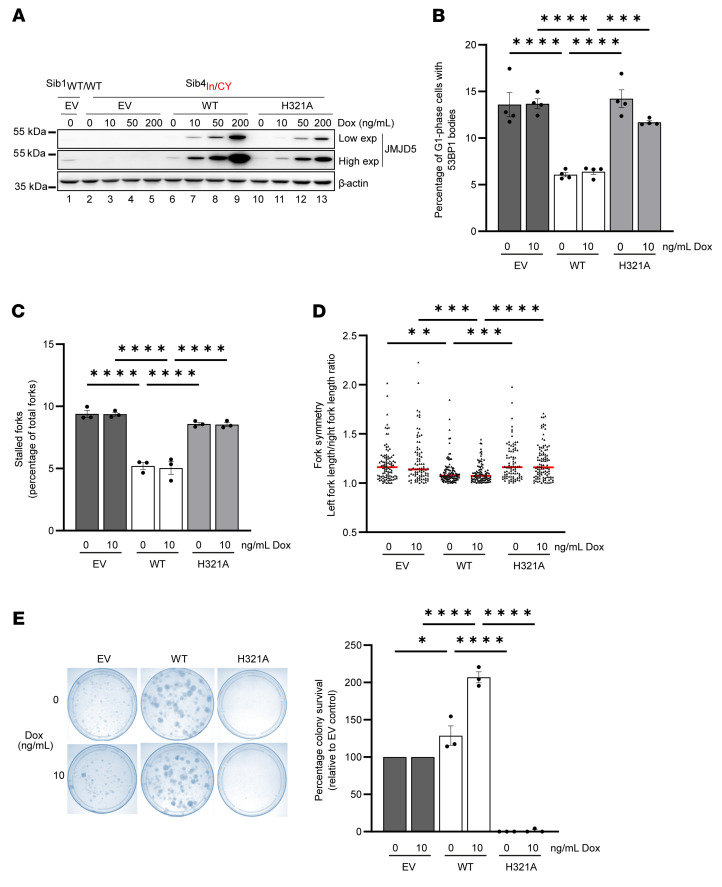
Decreased replication fidelity and viability of affected patient cells are due to loss of JMJD5 hydroxylase activity. Reconstitution of Sib4_In/CY_ fibroblasts with JMJD5. Sib4_In/CY_ cells were transduced with lentivirus encoding doxycycline-inducible WT or inactive (H321A) JMJD5 cDNA or a control empty vector (EV). Sib1_WT/WT_ cells were transduced with EV as a control. (**A**) Cells were treated with doxycycline for 14 days before Western blotting and immunofluorescence (Supplemental Figure 22A). “Low” and “High” refer to the exposure time. Note the leaky physiological re-expression of exogenous JMJD5 in the absence of doxycycline. (**B**–**E**) Because of modest heterogeneity in re-expression levels between cells (Supplemental Figure 22A), and slightly reduced expression of H321A compared with WT JMJD5, all subsequent experiments were conducted at 0 and 10 ng/mL doxycycline. Increased 53BP1 bodies (**B**), stalled replication forks (**C**), and replication fork asymmetry (**D**) in Sib4_In/CY_ cells are suppressed by JMJD5 hydroxylase activity. (**E**) Decreased colony survival of Sib4_In/CY_ cells is rescued by JMJD5 hydroxylase activity. Reconstituted Sib4_In/CY_ cells were plated at limited density and monitored for colony formation. Left: Example of stained colonies. Right: Quantification of colonies expressed as percentage survival relative to Sib4_In/CY_ EV control cells. In **B**, a minimum of 300 cells were counted per condition. In **C**, at least 200 forks were counted per sample. In **D**, at least 50 structures per condition were measured in each experiment. (**B**) Data represent mean ± SEM from 4 independent experiments. (**C**–**E**) Data represent mean ± SEM from 3 independent experiments. Statistical analyses used 1-way ANOVA with Tukey’s post hoc test (**B**, **C**, and **E**) or Kruskal-Wallis with Dunn’s correction (**D**); *P ≤ 0.05, **P ≤ 0.01, ***P ≤ 0.001, ****P ≤ 0.0001.

The association studies presented above are indicative of a role for impaired JMJD5 function in replication fidelity and cell survival. To test whether the observed phenotypes were dependent on JMJD5, we developed a reconstitution model in immortalized Sib4_In/CY_ fibroblasts using conditional JMJD5 expression vectors (Figure 6A and Supplemental Figure 22A). A catalytically dead mutant [H321A, Fe(II)-binding mutant] (Supplemental Figure 22, B and C) was included to test the dependence of any phenotypes on the hydroxylase activity of JMJD5. Initial expression trials with doxycycline dose titrations indicated that the system was “leaky,” with physiological expression of exogenous JMJD5 in the absence of doxycycline (Figure 6A, compare lane 6 with lane 1). These analyses also indicated that the H321A mutant was modestly underexpressed compared with wild-type JMJD5, which could be corrected by the addition of 10 ng/mL doxycycline to achieve comparable and physiological expression levels (Figure 6A, compare lane 11 with lanes 6 and 1). Therefore, we included doses of 0 and 10 ng/mL doxycycline in subsequent functional assays.

Consistent with a role for JMJD5 in protecting against spontaneous RS, reconstitution with wild-type JMJD5 (Supplemental Figure 23A) significantly reduced the increased level of spontaneous RS markers seen in cells derived from the affected patient, including G_1_-phase 53BP1 bodies (Figure 6B), micronuclei (Supplemental Figure 23B), stalled replication forks (Figure 6C), and replication fork asymmetry (Figure 6D). Importantly, reconstitution with catalytically inactive JMJD5 did not rescue these phenotypes (Figure 6 and Supplemental Figure 23).

Finally, reconstitution with wild-type JMJD5 increased the colony-forming ability of cells derived from the affected patient Sib4_In/CY_ (Figure 6E). Conversely, inactive JMJD5 not only failed to improve the colony survival of these cells, but strongly suppressed it (Figure 6E).

Overall, our data demonstrate, for the first time to our knowledge, that biallelic pathogenic JMJD5 variants are associated with an uncharacterized neurodevelopmental disorder that has not been previously described. Furthermore, we provide mechanistic evidence that loss of JMJD5 gives rise to increased levels of spontaneous RS, which is a direct consequence of reduced JMJD5 hydroxylase activity.

## Discussion

Here we have identified the arginyl hydroxylase *JMJD5* as a disease gene in humans and implicated its catalytic activity in the maintenance of normal DNA replication fidelity. To our knowledge, this is the first time that genetic variants in a member of the JmjC-only hydroxylase family of 2OG-oxygenases have been identified as the basis of a human disease. Importantly, this work also contributes to the emergence of protein hydroxylases as important regulators of human health and pathology (6, 8, 34).

Consistent with their role in the pathogenicity of the disorder described here, both inherited variants dramatically compromised the stability of JMJD5 protein (Figure 3). This was perhaps unsurprising for the protein product of the intronic variant due to a substantial deletion within the highly structured double-stranded β-helix fold of the catalytic domain. However, the effect of the C123Y missense variant was harder to predict, in part because the N-terminus of JMJD5 has not been well characterized either structurally or biochemically. Secondary structure and AlphaFold predictions suggest that C123 is located on the hydrophobic face of an amphipathic α-helix that forms part of a TPR domain (Figure 2D and Supplemental Figure 12). While the C123Y variant is predicted to be highly structurally disruptive to this fold, it nonetheless appears not to compromise other known functions of the JMJD5 N-terminus such as RCCD1 binding (Supplemental Figure 16), further suggesting that the N-terminal domain contains at least 2 functionally separable regions.

During our studies exploring potential associations between the biallelic JMJD5 genotype and cellular phenotypes, we identified increased RS in primary and immortalized fibroblasts derived from the affected patients in addition to a variety of other cell types (Figure 4, Supplemental Figures 19–21, and data not shown). Through reconstitution experiments, we demonstrated that the RS observed in cells with biallelic JMJD5 variants was due to reduced JMJD5 expression and activity (Figure 6). It is possible that the role of JMJD5 in normal replication fidelity explains, or at least contributes to, the clinical phenotype described. Disorders driven by pathogenic variants in replication machinery components are classically associated with severe developmental delay and microcephaly, and patient growth rarely responds to growth hormone therapy (35, 36). In contrast, the two affected patients identified here showed only a modest reduction in head circumference (–0.5 to 1.2 standard deviation [SD]), which, in the context of the severe developmental delay (weight –4 SD and height –6 to –7 SD), was therefore defined as relative *macro*cephaly. Furthermore, the growth velocity of both patients responded positively to hormone therapy (Supplemental Figure 2). In the future, it will be important to determine how consistently these clinical features are observed in other patients with pathogenic JMJD5 variants.

During revision of this article, we identified a new patient from an independent family (Family 2) with biallelic JMJD5/KDM8 variants and a clinical presentation that overlaps with the affected patients presented earlier (Family 1). Similarly to the affected patients from Family 1, the affected patient from Family 2 also presented with severe intrauterine growth retardation, failure to thrive, facial dysmorphism (including progeroid appearance), intellectual disability, microcephaly (but not relative macrocephaly), muscle hypotonia, and hair thinning (see Supplemental Figure 24A and Supplemental Patient Case Reports for full clinical description). This patient did not receive growth hormone replacement therapy. The compound heterozygous variants included a p.Asp293Asn (D293N) (c.877G>A) missense substitution inherited from the father and a p.Glu302Gly_fs*28 (E302Gfs*28) (c.905del) frameshift variant inherited from the mother (Supplemental Figure 24B and Supplemental Figure 25). The D293N variant is predicted by both FATHMM and PolyPhen to be damaging (scores of 0.99 and 1.00, respectively). The D293 residue is located within the hydroxylase domain (Supplemental Figure 26, A and C) and is entirely conserved (Supplemental Figure 11). The functional consequences of the D293N variant are difficult to predict, however, because it faces outward into the solvent rather than internally within the catalytic pocket (Supplemental Figure 26A). Indeed, the expression and in vitro activity of the D293N variant were largely normal (Supplemental Figure 26, D and E). Conversely, the E302Gfs*28 variant results in almost complete loss of the catalytic domain (Supplemental Figure 26, A and B). As with the JMJD5^Δ332–362^ variant of Family 1, attempts to express and purify recombinant E302Gfs*28 protein for hydroxylase assays were unsuccessful owing to very low yield and degradation. However, the E302Gfs*28 mRNA is likely degraded by nonsense-mediated decay, and structural analysis indicates that any residual protein would lack hydroxylase activity (Supplemental Figure 26, A and B). In the context of the results presented on Family 1, it is likely that the biallelic variants identified in Family 2 also underlie the neurodevelopmental disorder affecting this new patient. Although further work is required to understand the impact of these new variants on JMJD5 function, RS, and genome instability, the identification of an additional patient with an overlapping clinical presentation strengthens the assignment of JMJD5 as the causative gene in the disorders presented. Whether a similar neurodevelopmental disorder might arise as a consequence of inheriting pathogenic variants in genes encoding functional partners of *JMJD5*, such as *RCCD1*, is also of interest.

Although it is clear that JMJD5 plays a role in regulating DNA replication, it is not known whether this is direct or indirect. Severe or complete loss of JMJD5 expression through RNA interference or gene knockout approaches has been reported to lead to a variety of cellular phenotypes that could indirectly lead to RS, including defects in cellular proliferation (23, 37–40) and DNA damage repair (19, 23, 24). Although we did not observe any gross defects in proliferation or cell cycle progression in the hypomorphic JMJD5 patient-derived cell models described here (Supplemental Figure 17), we cannot definitively rule out their contribution to the RS phenotype. However, it is also possible that a direct role for JMJD5 in maintaining normal replication fidelity could underlie the previously reported associations of JMJD5 loss of function with cell proliferation and cell cycle progression, in addition to other processes such as transcriptional pausing (41). The increased prevalence of micronuclei and 53BP1 bodies in cells with reduced JMJD5 function suggests that its inhibition causes RS, which may be consistent with its being required for signaling or facilitating repair of DNA damage caused by RS.

Our reconstitution model provided an opportunity for structure/function experiments, specifically to test the hypothesis that the hydroxylase activity of JMJD5 is required for normal replication fidelity. We showed that, unlike the wild-type enzyme, an inactive Fe(II)-binding mutant of JMJD5 (H321A) was not able to restore normal replication fidelity or cell viability (Figure 6). Like other JmjC-only hydroxylases, JMJD5 has been assigned a number of enzyme activities that are still under debate (6, 10, 26) or require further investigation. These include histone demethylation (29, 42), histone proteolysis (43, 44), and protein hydroxylase activities (18, 45). Recent structural and biochemical studies suggest that JMJD5 is unlikely to demethylate or proteolyze histones (13, 17, 45, 46) or hydroxylate previously reported substrates and interactors (18). We also did not observe any JMJD5-dependent changes in histone H3 “clipping” or H3K36me2, RPS6, or p53 levels in patient-derived fibroblasts or HEK293T cells (Supplemental Figure 27). In relation to the reported role of JMJD5 in histone clipping and RNA polymerase I pausing (43), we did not observe any statistically significant difference in global chromatin accessibility around transcription start sites or mRNA expression of DDX3Y, KDM5D, UTY, or EIF2S3 in primary or immortalized Family 1 affected patient cells (Supplemental Figure 28). Lastly, we did not observe any activity-dependent interactions of JMJD5 with RPS6, histone H3, p53, or RCCD1 that might have been consistent with them being substrates (14, 16) (Supplemental Figure 27). We cannot exclude that such interactions or regulations were below the level of detection under the conditions of our experiments, however.

In summary, we have identified for the first time, to our knowledge, that *JMJD5* is a disease-associated gene, and investigated the cellular basis of the human phenotype associated with its pathogenic variants. In doing so, we have established a role for its hydroxylase activity in regulating DNA replication fidelity. Future studies investigating the role of JMJD5 in DNA replication may shed important new light on its role in normal physiology and human developmental disorders. This work raises the possibility that other Mendelian human disorders might be explained by genetic alterations in genes encoding related JmjC-only hydroxylases. Finally, it further supports the emergence of protein hydroxylases and the 2OG-oxygenase family as important regulators of fundamental cellular processes and drivers of human pathology.

## Methods

Further information can be found in Supplemental Methods.

### DNA expression vectors.

An N-terminally HA epitope–tagged JMJD5 expression vector was generated by cloning of the human *JMJD5* open reading frame (ORF) into pEF6 (a gift from Richard Marais, Cancer Research UK Manchester Institute, Manchester, UK) by PCR using Phusion High Fidelity DNA Polymerase (New England Biolabs). Doxycycline-inducible JMJD5 expression vectors (untagged and N-terminally FLAG tagged) were created by PCR subcloning into modified versions of the lentiviral vectors pTRIPZ (Dharmacon), in which the RFP cDNA was replaced with a modified multiple cloning site (termed pTIPZ; a gift from Matthew Cockman, Francis Crick Institute, London, UK), and pGIPZ, in which the GFP cDNA was also replaced with a limited multiple cloning site (termed pIPZ; a gift from Tencho Tenev, Institute of Cancer Research, London, UK). The *JMJD5* ORF was also PCR subcloned into pGEX-4T-1 (Amersham) for production of N-terminally glutathione S-transferase–tagged (GST-tagged) recombinant protein in *E*. *coli*. pEF6 HA-JMJD5 was used as a template for site-directed mutagenesis PCR reactions using custom non-overlapping HPLC-purified primers phosphorylated with T4 polynucleotide kinase (Thermo Fisher Scientific Phusion Site-Directed Mutagenesis kit). PCR templates were removed by DpnI digestion and products purified (GenElute, Sigma-Aldrich) before transforming into DH5α. Successful mutagenesis was verified by Sanger sequencing before subcloning into the vectors described above. The pBABE-puro-hTERT human telomerase expression vector used to immortalize primary dermal fibroblasts was from Addgene (plasmid 1771).

### Cell treatments.

siRNA transfections were carried out using Lipofectamine RNAiMAX (Invitrogen), according to the manufacturer’s instructions. All siRNAs were used at a working concentration of 30 nM. siRNA oligonucleotides were from Sigma-Aldrich (Supplemental Figure 29). Control transfections were carried out using a negative control siRNA (Sigma-Aldrich SIC001). Cells were analyzed 72 hours after transfection. DNA plasmid transfection was performed using FuGENE (Promega) according to the manufacturer’s instructions. Conditional expression of cDNA was induced using doxycycline, 24 hours after initial transfection or after plating of stable cell lines. The concentration and duration of doxycycline incubation are indicated for each experiment. To monitor protein stability or proteasomal degradation, cells were treated with cycloheximide (50 μg/mL) or MG132 (10 μM), respectively. Where indicated, cells were treated with 1 mM hydroxyurea or 0.5 μM or 0.1 μM aphidicolin (Sigma-Aldrich).

### Cell culture and formation of cell lines.

Dermal fibroblasts were isolated as follows: A small piece of the upper layer of skin from the internal side of the forearm was excised using tweezers. Skin tissue was then cut into small pieces and fibroblasts expanded in RPMI plus Amniomax (Thermo Fisher Scientific). HeLa (CLL-2) and HEK293T (CRL-3216) cell lines were purchased from ATCC. MCF10A cells were a gift from Clare Davies (University of Birmingham). HeLa and HEK293T cells were grown in DMEM plus 10% (vol/vol) FBS (Sigma-Aldrich). MCF10A cells were cultured in DMEM/F12 (1:1) supplemented with 5% horse serum, 20 ng/mL EGF, 0.5 μg/mL hydrocortisone, 100 ng/mL cholera toxin, 10 μg/mL insulin. All cells were cultured at 37°C, in a humidified atmosphere, with 5% CO_2_ and media supplemented with penicillin and streptomycin (Gibco). Cells were routinely tested for mycoplasma (LookOut Mycoplasma PCR Detection Kit, Sigma-Aldrich). Fibroblasts were immortalized with a retrovirus expressing human telomerase reverse transcriptase, as follows: Retroviral supernatant was generated by cotransfection of HEK293T cells with the human telomerase retroviral expression vector and viral packaging vectors pCMV-Gag-Pol and pMD2.G using FuGENE. Forty-eight hours after transfection, virus-containing medium was passed through a 0.45 μm filter onto primary dermal fibroblasts. Transduced fibroblasts were selected and grown in DMEM plus 20% (vol/vol) FBS and 70 μg/mL hygromycin. To generate lentivirally transduced stable cell lines, HEK293T cells were cotransfected using FuGENE with lentiviral vectors expressing doxycycline-inducible JMJD5 cDNA (details above) plus psPAX2 and pMD2.G packaging vectors. Filtered lentiviral supernatant was incubated with recipient cells before selection and growth of transduced cell lines in 1 μg/mL puromycin.

### Cell proliferation and colony formation assays.

Cell proliferation/viability was measured using an MTS assay (CellTiter 96 Aqueous One Solution Cell Proliferation Assay, Promega). Cells were incubated in MTS working reagent (1:20 ratio of phenazine methosulfate to CellTiter 96 Aqueous MTS reagent dissolved in PBS) for 1 hour at 37°C protected from light; then absorbance was measured at 490 nm using an EnSpire plate reader (PerkinElmer). Colony formation was performed using limited dilution (e.g., 200 cells per well of a 6-well plate). Two weeks after seeding, fibroblast colonies were fixed and stained using 0.1% (wt/vol) crystal violet (Fluka) in 50% methanol.

### Cell cycle analysis.

Cell cycle analysis was performed on fibroblasts fixed in ice-cold 70% ethanol. Cells were incubated with 100 μg/mL RNase A (Thermo Fisher Scientific) and stained with 20 μg/mL propidium iodide, then analyzed using a CyanB flow cytometer and Summit v4.3 software (Beckman Coulter).

### Quantitative PCR.

RNA was purified using the GenElute Mammalian Total RNA Miniprep Kit (Sigma-Aldrich) and cDNA generated using the High Capacity cDNA Reverse Transcription Kit (Applied Biosystems). Quantitative PCR (qPCR) was then performed using the manufacturer’s protocol for the PCR Master Mix (Applied Biosystems). Transcript abundance was measured using a JMJD5-specific FAM-labeled TaqMan probe (Hs00227070_m1) and an internal loading control (VIC-labeled *ACTB* probe; 4326315E) (Thermo Fisher Scientific). qPCR was performed on a QuantStudio 5 Real-time PCR System (Thermo Fisher Scientific), and the comparative 2^–ΔΔCt^ method was used to quantify transcript abundance.

### Western blotting.

Whole cell extracts were prepared in JIES (100 mM NaCl, 20 mM Tris-HCl pH 7.4, 5 mM MgCl_2_, 0.5% [vol/vol] NP-40) or RIPA buffer (150 mM NaCl, 25 mM Tris pH 8, 1% [vol/vol] NP-40, 0.5% [vol/vol] sodium deoxycholate, 0.1% [vol/vol] SDS) supplemented with 1× SIGMAFAST protease inhibitor cocktail (Sigma-Aldrich). Samples were separated by SDS-PAGE using 12% (vol/vol) polyacrylamide gels. Proteins were transferred onto PVDF membranes (GE Healthcare), and immunoblotting was performed using the indicated antibodies (Supplemental Figure 29). All antibodies were from commercial sources apart from anti-JMJD5 antibodies: Mouse anti-JMJD5 was a gift from Matsuura Yoshiharu (Osaka University, Osaka, Japan), and rabbit anti-JMJD5 was custom-made by immunization with JMJD5 peptide (Bethyl Laboratories). Chemiluminescent signal was visualized using Clarity or Femto ECL (Bio-Rad) and detected by a Fusion FX Vilber Imager. For Coomassie staining, gels were incubated in InstantBlue (Sigma-Aldrich).

### Recombinant JMJD5 protein.

pGEX-4T-1 JMJD5 was transformed into BL21(DE3)-competent cells (Promega). Overnight cultures were grown in 2× Luria-Bertani (LB) broth plus 50 μg/mL ampicillin, then diluted 1:100 in 1 L of room temperature 2× LB plus 50 μg/mL ampicillin. Cultures were incubated at 37°C and 200 rpm until an absorbance of 0.6 at OD_600nm_. Cultures were chilled to 18°C before induction of protein expression with 0.5 mM IPTG for 16 hours. Bacterial pellets were resuspended in 100 mL lysis buffer (50 mM Tris pH 8.0, 300 mM NaCl, 0.5 mM TCEP [tris(2-carboxyethyl)phosphine; Sigma-Aldrich], 1× SIGMAFAST protease inhibitor, 20 U/mL TurboNuclease [Sigma-Aldrich], 0.5 mg/mL lysozyme [Sigma-Aldrich], and 0.1% [vol/vol] Triton X-100) and rotated for 2 hours at 4°C. Samples were centrifuged at 25,000*g* for 15 minutes at 4°C. Supernatants were rotated at 4°C overnight with 1 mL Glutathione Sepharose beads (GE Healthcare). Beads were washed 6 times with wash buffer (50 mM Tris pH 8.0, 300 mM NaCl, 0.5 mM TCEP, and 0.1% [vol/vol] Triton X-100) before elution of GST-tagged JMJD5 with 10 mM glutathione (Sigma-Aldrich), 50 mM Tris (pH 8.0), 300 mM NaCl, and 0.5 mM TCEP. Three sequential 1 mL elutions were performed at room temperature for 10 minutes with rotation. Elutions were combined, then dialyzed overnight at 4°C in a 7,000-molecular-weight-cutoff Slide-A-Lyzer dialysis cassette (Pierce) against 50 mM Tris (pH 8.0), 300 mM NaCl, 0.5 mM TCEP. Dialyzed samples were concentrated using a 10,000-molecular-weight-cutoff protein concentrator (Pierce).

### In vitro interaction assay.

Glutathione Sepharose beads were preblocked with 5% (wt/vol) BSA (Sigma-Aldrich) for 2 hours rotating at 4°C before being washed in JIES and incubated with HEK293T whole-cell lysate and either 6 μg GST, GST-tagged wild-type JMJD5, or GST-tagged JMJD5 C123Y. Samples were rotated at 4°C for 2 hours before beads were washed 6 times in JIES and then boiled in 2× Laemmli buffer for 5 minutes. Samples were analyzed by Western blotting and Coomassie staining.

### In vitro hydroxylation assay.

A 400 μM stock of RPS6 peptide (^129^VPRRLGPKRASRIRKL^144^) was prepared in substrate buffer (50 mM Tris-HCl pH 7.5, 150 mM NaCl, 1 mM DTT). (NH_4_)_2_Fe(SO_4_)_2_ was prepared in 20 mM HCl to a concentration of 500 mM and then diluted to 10 mM with MilliQ water before use. A 2× master mix of buffers and cofactors was prepared as follows: 50 mM HEPES (pH 7.5), 200 μM Fe(II), 1 mM ascorbate, and 400 μM 2-oxoglutarate. Hydroxylation reactions comprised 10 μL 2× cofactor mix, 20 μg GST-tagged wild-type, H321A, D293N, or C123Y JMJD5, and 5 μL of substrate buffer or RPS6 peptide (total volume 20 μL). Reactions were incubated at 37°C for 1 hour before quenching in 1% formate. Hydroxylation activity was analyzed using the Succinate-Glo JmjC demethylase/hydroxylase assay (Promega) according to the manufacturer’s instructions. Luminescence was detected using a PerkinElmer Enspire plate reader.

### Immunofluorescence.

Cells grown onto glass coverslips were fixed with 4% (vol/vol) paraformaldehyde for 15 minutes at room temperature. For 53BP1 staining, soluble protein was removed before fixation using pre-extraction buffer (20 mM NaCl, 3 mM MgCl_2_, 300 mM sucrose, 10 mM PIPES, 0.5% [vol/vol] Triton X-100) on ice for 5 minutes. Cells were permeabilized using 0.1% (vol/vol) Triton X-100 for 10 minutes at room temperature and blocked in 1% (wt/vol) BSA (Sigma-Aldrich) in PBS for 1 hour at room temperature. Coverslips were incubated with indicated antibodies (Supplemental Figure 29), then washed and incubated with DAPI (Invitrogen) for 10 minutes at room temperature before mounting with ProLong Gold (Cell Signaling). Cells were imaged using a Leica DM6000B or Zeiss Axio Observer microscope.

### DNA fiber assay.

Progression of DNA synthesis was monitored with the DNA fiber assay using halogenated thymidine analogs CldU and IdU (Sigma-Aldrich). Cells were incubated at 37°C with 25 μM CldU for 20 minutes, washed with PBS, then treated with 1 mM hydroxyurea for 2 hours at 37°C before incubation with 250 μM pre-equilibrated IdU for 20 minutes. Untreated control cells were incubated sequentially with each analog for 20 minutes. Cells were collected by trypsinization and washed in ice-cold PBS. Cells (5 × 10^5^) were lysed in spreading buffer (200 mM Tris pH 7.4, 50 mM EDTA, 0.5% [wt/vol] SDS) directly on microscope slides and fixed in methanol/acetic acid (3:1 ratio). Denaturation with 2.5 M HCl preceded immunofluorescence staining to detect the thymidine analogs. After incubation in primary antibodies (Supplemental Figure 29), slides were fixed with 4% (vol/vol) paraformaldehyde for 10 minutes before incubation with secondary antibodies (Supplemental Figure 29). DNA fibers were imaged with a Leica DM6000B fluorescence microscope using ×40 magnification and analyzed using ImageJ (NIH) or LasX software (Leica). Replication fork asymmetry was measured by calculation of the ratio between the lengths of the IdU tracts present in first-label origin structures. Replication fork speeds were calculated by measurement of CldU and IdU tract lengths in ongoing forks using the conversion 1 μm corresponds to 2.59 kb.

### Statistics.

Statistical analyses were performed on experiments with at least 3 independent biological repeats, and the statistical test used is indicated for each experiment. GraphPad Prism was used to perform 1-way ANOVA and Kruskal-Wallis tests.

### Protein sequence conservation and structural modelling.

Primary sequence conservation was analyzed using the PRALINE server (www.ibi.vu.nl/programs/pralinewww/) with default settings. 3D structural prediction was undertaken using the SYMPRED server (www.ibi.vu.nl/programs/sympredwww/). Tertiary structure prediction used LOMETS (https://zhanggroup.org/LOMETS/) and I-TASSER (https://zhanglab.ccmb.med.umich.edu/I-TASSER/). Visualization of the region deleted in the JMJD5^Δ332–362^ and E302Gfs*28 variants was performed by annotation of the JMJD5 crystal structure (Protein Data Bank: 4GAZ) using UCSF Chimera (https://www.cgl.ucsf.edu/chimera/). A structural model of the JMJD5 N-terminus was obtained from AlphaFold (H3BM39).

### Study approval.

The study was conducted in accordance with the Declaration of Helsinki, and approval was granted by the Tartu University Ethics Committee (Certificates 259/T-2, 263/M-16, 283/M-10, and 287/M-15). Informed consent was obtained from all family members from Families 1 and 2 (Certificates 259/T-2, 263/M-16, 283/M-10, and 287/M-15 for Family 1). Written informed consent for publication of the photographs of affected siblings was obtained separately from the parents.

## Author contributions

SCF, CH, and MLC wrote the manuscript with contributions and revisions by all authors. TJK contributed to replication stress analyses, and performed all CENPA/53BP1 staining and analyses of micronuclei. SP was responsible for the analytical aspects of the Estonian clinical work. KÕ, SP, and MHW analyzed genome sequencing data for Family 1. VT is a child endocrinologist who was responsible for treatment and growth data analysis of Family 1 patients. KÕ is the main clinical geneticist for Family 1 and took skin biopsies and obtained all consent needed. DAC analyzed and interpreted the genomic information for Family 2. GSS established primary patient-derived fibroblasts. GSS and MRH provided expert input and training in replication stress analyses and data interpretation. SCF and CH performed all the in vitro and in vivo experiments. UB contributed to project molecular biology and genotyping of patient-derived fibroblasts. TJK analyzed protein sequence conservation under guidance from CAEW. CL and SJS performed and interpreted protein bioinformatics including structural modeling. KTO built figures illustrating patient height and weight. JP and RR are responsible for the primary care and description of medical history and phenotypic characteristics of the patient from Family 2. MS, EH, RA, and UB cloned and analyzed the patient variants from Family 2. SKP contributed to analyses of primary fibroblast JMJD5 expression. Genomic analyses were performed under the supervision of ADB, with ATAC-Seq delivered by AP and bioinformatic analyses by MEEA.

## Supplementary Material

Supplemental data

## Version 1 02/16/2023

In-Press Preview

## Version 2 04/03/2023

Electronic publication
