# Supplementary material for: Impaired protein hydroxylase activity causes replication stress and developmental abnormalities in humans
Source: J Clin Invest. 2023 Apr 3;133(7):e152784. doi: 10.1172/JCI152784 (PMC10065073; doi:10.1172/JCI152784)
Supplement: Supplemental data [file jci-133-152784-s028.pdf]

Supplemental Material

Sally C Fletcher[1], Charlotte Hall[1], Tristan J Kennedy[1], Sander Pajusalu[2,3], Monica H Wojcik[4,5], Uncaar Boora[1], Chan Li[1], Kaisa Teele Oja[2,3], Eline Hendrix[1], Christian A E Westrip[1], Regina Andrijes[1], Sonia K Piasecka[1], Mansi Singh[1], Mohammed E El-Asrag[1,6], Anetta Ptasinska[1], Vallo Tillmann[7,3], Martin R Higgs[1], Deanna Alexis Carere[8], Andrew D Beggs[1], John Pappas[9], Rachel Rabin[9], Stephen J Smerdon[1], Grant S Stewart[1], Katrin Õunap[2,3], Mathew L Coleman[1,10]

[1]Institute of Cancer and Genomic Sciences, University of Birmingham, Birmingham, UK
[2]Department of Clinical Genetics, Genetics and Personalized Medicine Clinic, Tartu University Hospital, Tartu, Estonia
[3]Institute of Clinical Medicine, University of Tartu, Tartu, Estonia
[4]Center for Mendelian Genomics, Broad Institute of MIT and Harvard, Cambridge, Massachusetts, USA
[5]Divisions of Newborn Medicine and Genetics and Genomics, Department of Pediatrics, Boston Children's Hospital and Harvard Medical School, Boston, MA, 02115
[6]Faculty of Science, Benha University, Benha, Egypt
[7]Children’s Clinic, Tartu University Hospital, Tartu, Estonia
[8]GeneDx, Gaithersburg, MD, 20877, USA
[9]Clinical Genetic Services, Department of Pediatrics, NYU Langone Medical Center, New York, NY.

[10]To whom correspondence should be addressed m.coleman@bham.ac.uk,
Telephone: +441214143943

Conflict of interest: The authors have declared that no conflict of interest exists.

Disclosure: D.A.C. is an employee of GeneDx, LLC.

Supplementary Patient Case Reports

Family 1 - history

Two elder brothers ($Sib1_{WT/WT}$ and $Sib3_{In/WT}$), one sister ($Sib2_{In/WT}$) and father ($Pat_{In/WT}$) are healthy (Figure 1B). The mother ($Mat_{WT/CY}$) had one miscarriage and was diagnosed with breast cancer at the age of 42. The clinical presentation of the affected siblings are detailed below, and summarized in Supplemental Figure 1. Calculation of growth s.d. used Estonian growth curves (https://www.ravijuhend.ee/uploads/userifles/Lapse_tervise/lapse-tervise-rj-a5-v1_lisa-4.pdf). The father and mother are relatively tall at 190 cm and 171 cm, respectively. Therefore, the target heights for $Sib4_{In/CY}$ and $Sib5_{In/CY}$ were set at +0.8 s.d. and +1.0 s.d., respectively.

Family 1 - Male affected sibling ($Sib4_{In/CY}$)

Fetal hypotrophy was diagnosed at 33 weeks of pregnancy. The child was born at term with asymmetrical failure to thrive (weight 2.36 kg (-3 s.d.), length 43 cm (-3.5 s.d.) and occipitofrontal circumference (OFC) 33 cm (-2 s.d.)). Apgar score was 8/9. Dysmorphic features were noted including high forehead, micrognathia, dysmorphic ears, bilateral simian crease, hypospadias, sacral dimple and an L-shape right kidney. At two months, he had a brain contusion with subdural hematoma and frontal bone fracture, from which he recovered normally. Initial analyses identified a normal karyotype (46, XY) and metabolic parameters.

At 22 months there was severe failure to thrive (height 65 cm (-5.5 s.d.), weight 5.66 kg (-6 s.d.) and OFC was 44 cm (-3.5 s.d.)). Dysmorphic features included triangular face, high forehead, broad and high nasal bridge, broad nasal tip, large mouth, high palate, micrognathia, dysplastic ears, short neck, bilateral simian crease, F5 clinodactyly, hypospadias, sacral dimple, and a right club foot. Development was delayed: He sat and crawled but did not stand or walk. He had muscular hypotonia and excessive sweating. It was difficult to evaluate whether delayed development was caused by a genetic disorder or the consequence of the earlier brain trauma. He had frequent bronchitis during his first year. Bone

age was delayed and corresponded to a 9 month old. Brain MRI showed mild brain atrophy. Ophthalmology diagnosed left-sided hyperopia and astigmatism.

Endocrine investigation included two growth hormone (GH) stimulation tests. The maximum peak GH response was 9.1 µg/L suggesting partial GH deficiency. This was confirmed by low IGF-1 levels of 43.7 µg/l (the normal range is 49-289). TSH, fT4, prolactin and cortisol hormone levels were in the normal range. GH therapy was started at the age of 2 years and 2 months. The treatment was discontinued after 21 months because the response was only modest (growth velocity increased from 6.0 cm/year to 7.0 cm/year). However, during the treatment his muscular tone improved and his sweating was reduced. Because his growth velocity fell significantly over the two years following discontinuation of GH treatment (to 2.2 cm /year), treatment with GH was restarted at the age of 6.6 years. This second treatment elicited a good growth response (Supplemental Figure 2).

The clinical presentation at the age of 22 months had some similarities to Silver-Russell syndrome (four points by the Netchine–Harbison clinical scoring system) (1). However, analyses of molecular defects associated with that condition, including hypomethylation of the 11p15 chromosomal region and UPD7/14 molecular tests, were normal. Although a chromosomal microarray analysis revealed a 400-kb duplication in the region of 11q25 (Hg 36, 11:133,817,698-134,227,062), this duplication was not present in the younger affected sibling or their parents. Multiplex PCR assays covering relevant regions of chromosomes 6, 7, 11 and 14 associated with congenital imprinting disorders were negative.

At the age of 3.5 years, he was taking some steps with assistance. Although he could not speak any words, he was using some syllables. His height was 74 cm (-5.5 s.d.), his weight was 7.4 kg (-5.5 s.d.), and his OFC was 49.5 cm (-1 s.d.). Hypoplastic mammillaes were noticed in addition to the previously observed dysmorphic features. At this time, the family were discussed in the Dysmorphology Session of the European Society of Human Genetics meeting, 2010. The main suggestion was to suspect an atypical variant of Coffin-Siris syndrome or Mabry syndrome. Mabry syndrome was excluded by the absence of hyperphosphatasia. Because the etiology of Coffin-Siris syndrome was not known at that time,

he was not tested further in that regard. However, the subsequent identification of genes involved in Coffin-Siris syndrome supported an analysis of patient WGS data, which was negative (Supplemental Figure 3).

At the age of 11 years, GH treatment was discontinued for 6 months due the deterioration of his scoliosis. He had moderate intellectual disability and autistic features and was studying in a school for disabled children. Although he had no speech, he was saying a few words and understood commands. GH therapy increased his physical activity and strength. He still suffered from frequent upper respiratory infections.

Family 1 - Female affected sibling ($Sib5_{In/CY}$)

The mother was evaluated carefully up to the 22nd week of pregnancy in the department of clinical genetics at Tartu University Hospital. Although ultrasound investigations and screening tests were normal, fetal hypotrophy was diagnosed in the third trimester. She was born at term with low birth weight (2.39 kg (-2.5 s.d.)), length (43 cm (-3.5 s.d.)) and OFC (33.5 cm (-1.5 s.d.)), and had Apgar scores of 6/7/7. She presented with dysmorphic features included high and prominent forehead, deep set eyes, high nasal bridge, microretrognathia, dysplastic ears, and abnormal position of fingers and toes. At 2 months of age the following were also noted; short neck, short hands and feet, dry skin, hypoplastic mammillae's and F5 clinodactyly.

At the age of 15 months, she had extreme failure to thrive, with only 0.5 kg of weight gain during her first year of life. Her weight was 3.91 kg (-6 s.d.), her height 55 cm (-7.5 s.d.), and her OFC 45 cm (-2 s.d.). She had the same dysmorphic phenotype as previously characterized. She was able to hold her head up, turn and stand. She was able to babble to communicate. Her development corresponded to 6-10 months by the Griffith scale. A brain ultrasound showed wide lateral ventricles. Her muscular tone was hypotonic. She had good appetite. Her bone age was delayed and corresponded to 3 months of age. From 22 months of age she had repeatedly elevated serum TSH and low normal free T4 levels (9.42 – 9.57 mU/L and 12.1-13.2 pmol/L respectively). At 3.5 years of age treatment with L-Thyroxine was started. As she did not show increased growth, GH treatment was started at the age of 4

years. The response to the treatment was good: growth velocity increased from 4.5 cm /year to 7.0 cm/year (see Supplemental Figure 2).

Her mental development was better than her elder affected brother, but mild intellectual disability was diagnosed. A brain MRI showed mildly dilated ventricles. She had strabismus and hyperopia. It was noted that she had an unusually deep voice. She also had frequent upper respiratory infections, similar to her elder affected brother. At the age of 8 years, she also started to study at a school for disabled children.

As noted above, both Family 1 affected patients had particularly low weight s.d. Over the course of their primary care of the Family 1 several X-ray examinations of the spine, pelvis, head and extremities were required. However, no signs of inherited bone disease were noted.

Family 2 - Affected male ($Sib1_{E302fs*28/D293N}$)

The pregnancy was complicated by pre-eclampsia and severe intra-uterine growth retardation. The patient was born at 27 weeks and 6 days of gestation and weighed 770 grams. The patient was in the neonatal intensive care unit for approximately 4 months. He required intubation for approximately 1 month and received a gastrostomy tube. The patient had chronic lung disease due to prematurity. The gastrostomy tube was removed at 15 months of age. He continued to have feeding difficulties, with vomiting and oral aversions. At 3 years old he was not eating solid foods. The patient has persistent microcephaly and failure to thrive: At 3 years of age, his height was 84.1 cm and weight was 9.6 kg. His head circumference was 46.5 cm at 32 months. Clinical evaluation at 2 years 5 months was significant for progeroid appearance with periorbital fullness. He also has hair thinning. The patient first walked at 21 months of age. At 3 years old, he was babbling, but did not have any words. The patient was noted to have muscular hypotonia. He had a history of a simple febrile seizure at 2 years 4 months. He has not yet received brain imaging or electroencephalograms. At 2 years 5 months, the patient underwent trio exome sequencing at GeneDx and was found to be compound heterozygous for a paternally inherited variant c.877 G>A p.Asp293Asn and a maternally inherited variant c.905del p.Glu302Glyfs*28 in the KDM8/JMJD5 gene. Microarray analysis identified a de novo

duplication variant at 15q21.3 (54888930_55744654) of unknown clinical significance. The family history was unremarkable.

Supplementary Methods

Genomic Analyses

Family 1 - Whole genome sequencing and data processing were performed by the Genomics Platform at the Broad Institute of MIT and Harvard. PCR-free preparation of sample DNA (350 ng input at >2 ng/ µL) was accomplished using Illumina HiSeq X Ten v2 chemistry. Libraries were sequenced to a mean target coverage of >30x. Genome sequencing data was processed through a pipeline based on Picard (broadinstitute.github.io/picard/), using base quality score recalibration and local realignment at known insertions/deletions (indels). The BWA aligner was used for mapping reads to hg38. Single Nucleotide Variants (SNVs) and indels were jointly called across all samples using Genome Analysis Toolkit (GATK) HaplotypeCaller package version 3.4. Default filters were applied to SNV and indel calls using the GATK Variant Quality Score Recalibration (VQSR) approach. Annotation was performed using Variant Effect Predictor (VEP). Lastly, the variant call set was uploaded to seqr for collaborative analysis between the CMG and investigator.

Data sharing statement from the Broad Institute Center for Mendelian Genomics (CMG)

The Broad Institute CMG shares raw sample genotype data (crams), phenotype data (high level and HPO terms), and causal and candidate gene/variant information. Raw sequence data is uploaded to AnVIL (https://anvilproject.org/data/) one year after it is released to the CMG collaborator. Data access applications are through dbGaP (http://www.ncbi.nlm.nih.gov/gap) Study Accession phs001272.v1.p1. Data in this manuscript can be found in project: AnVIL_CMG_Broad_Orphan_Estonia-Ounap_WGS. Phenotype data and candidate gene/variant information are shared through seqr with the Matchmaker Exchange system (https://www.matchmakerexchange.org/). All rare variants are also

uploaded to Geno2MP (http://geno2mp.gs.washington.edu), which is a web-based query tool that searches a database of rare variants from exome sequencing data linked to phenotypic information from a wide variety of Mendelian gene discovery projects like the CMGs. All candidate disease-gene relationships are posted publicly on the CMG website (http://mendelian.org/) within 19 months of discovery, with stronger candidates released within 13 months. Finally, causal variants are shared to ClinVar (http://www.ncbi.nlm.nih.gov/clinvar/) on an ongoing basis. Published variants are submitted to ClinVar within three months of publication.

Family 2 - Using genomic DNA from the proband and parents, the exonic regions and flanking splice junctions of the genome were captured using the IDT xGen Exome Research Panel v1.0 (Integrated DNA Technologies, Coralville, IA). Massively parallel (NextGen) sequencing was done on an Illumina system with 100bp or greater paired-end reads. Reads were aligned to human genome build GRCh37/UCSC hg19, and analyzed for sequence variants using a custom-developed analysis tool. Raw sequence data were uploaded to the NCBI SRA database under BioProject accession number PRJNA930714. Reported variants were confirmed, if necessary, by an appropriate orthogonal method in the proband and, if submitted, in selected relatives. The general assertion criteria for variant classification are publicly available on the GeneDx ClinVar submission page (http://www.ncbi.nlm.nih.gov/clinvar/submitters/26957/).

Sanger sequencing - The presence of the *JMJD5* variants was also confirmed in immortalized dermal fibroblasts at the genomic DNA and mRNA level. Genomic DNA was purified from cell pellets using the Invitrogen K1820-01 kit. Alternatively, RNA was purified using the GenElute Mammalian Total RNA Miniprep kit (Sigma-Aldrich) and cDNA generated using the High Capacity cDNA reverse transcription kit (Applied Biosystems). PCR reactions were performed using custom primers (Supplemental Figure 28) on template cDNA or genomic DNA. In order

to Sanger sequence the intronic variant, products from genomic PCR reactions were TOPO cloned (ThermoFisher) and transformed into DH5α competent *E.coli* (NEB). DNA purified from bacterial colonies was then screened by Sanger sequencing (Source Bioscience) to identify variants.

Assay for transposase-accessible chromatin with high-throughput sequencing (ATAC-seq) - Chromatin accessibility was evaluated by ATAC-seq using a modified protocol as reported (2, 3). ATAC-seq was performed on 50,000 cells per sample. Transposition reaction was performed for 30 min at 37°C upon addition of 45 µL of ATAC reaction mix, consisting of 25 µL Tagmentation DNA Buffer and 2.5 µL Tn5 Transposase enzyme (both from the Nextera DNA Library Prep Kit), 1 µL of 0.5% Digitonin and 16.5 µL water. DNA was purified using a MinElute Reaction Cleanup Kit and DNA fragments were subsequently amplified using Customized Nextera PCR Primer Adaptors and NEBNext High-Fidelity 2x PCR Master Mix. Optimal number of cycles, prior reaching saturation of the PCR in order to reduce GC and size bias, was determined by monitoring the reaction as previously described (2). Adaptor dimers were cleaned up from the libraries using AMPure magnetic beads prior to validation. Libraries were evaluated using a High Sensitivity DNA chip on an Agilent Technologies 2100 Bioanalyser instrument and concentration was measured using a KAPA Library Quantification Kit. Libraries were sequenced in a pool of twelve indexed libraries in a NextSeq (Illumina) machine and a NextSeq® 500/550 High Output 150 cycle sequencing kit v2, obtaining 75 bp paired-end reads, at the Genomics Birmingham sequencing facility. ATAC-seq reads were trimmed to remove adapter sequence using cutadapt (v. 4.1) (https://cutadapt.readthedocs.io/en/stable/) with the parameter –minimum-length = 20. Reads were then aligned to the GRCh38 reference genome (Ensembl release 103) using BWA (v. 0.7.17) (4). The data then processed in Sam/bam format using Samtools (v. 1.16.1), PCR duplicates and those aligned to the mitochondrial genome were removed using PicardTools (v. 2.27.4). ATAC-seq peaks were called on aligned and filtered BAM files using MACS2 (v. 2.2.7) (7). Generation of gene-body meta-profile from bigWig files, calculation genome-wide

enrichment and plotting of data profile for scores over sets of the genomic regions were performed using deepTools (5).

RNA-seq - RNA from patient-derived fibroblasts was extracted using the Sigma GenElute Mammalian Total RNA Miniprep kit. Library preparation was performed using a Lexogen QuantSeq 3' mRNA-Seq Library Prep Kit FWD for Illumina (Lexogen). Sequencing was performed using a NextSeq High 75 v2.5 flow cell on the NextSeq 500 (Illumina) NGS platform using 100 bp single-end reads. Raw sequencing data for all samples in Fastq format gave a minimum of 12.1×10^6 reads (average 15.4×10^6). The quality was checked using FastQC tools, trimming of the adaptors and removing sequencing reads with quality scores less than 20 were performed using Trim_Galore! (v 0.6.6). The Fastq files were then aligned to the human reference genome sequence (GRCh38) using STAR aligner version 2.7.8a with the outSAMtype BAM SortedByCoordinate option (6). Gene and exon level counts were generated from the binary alignment map (BAM) files using the Python library HTSeq (v 2.0.2) (7) and the featureCounts function (8) in the Rsubread package (v 1.26.1) in R with Homo_sapiens.GRCh38.103.gtf from the Ensembl database. Count normalization and differential exon usage using RNA-seq exon counts was performed using DEXSeq (1.36.0) (7). Figures were made in R using ggplot2 (v3.3.6) and Gviz (v 1.34.1) packages.

Bioinformatics analysis

The potential pathogenic nature of the C123Y and D293N *JMJD5* variants were predicted using SIFT (https://sift.bii.a-star.edu.sg/), PolyPhen-2 (http://genetics.bwh.harvard.edu/pph2/), and/or FATHHM (http://fathmm.biocompute.org.uk/). To establish evolutionary conservation JMJD5 protein sequences from different species were aligned using Clustal Omega multiple sequence alignment software (https://www.ebi.ac.uk/Tools/msa/clustalo/). To predict the potential effect of the eight-base-pair intron variant on *JMJD5* gene splicing, Human Splice Finder (HSF) version 3.1 was

utilized (http://www.umd.be/HSF/). The molecular weight and isoelectric point of wildtype and ∆332-362 JMJD5 were analyzed using the online software ExPASy (https://www.expasy.org/).

Supplemental Figure 1

Patient		$Sib4_{In/CY}$	$Sib5_{In/CY}$
**Primary clinician**		Katrin Õunap, Tartu	Katrin Õunap, Tartu
**Gene**		*KDM8*	*KDM8*
**Variant 1**		NM_024773: c.482G>A (p.Cys123Tyr)	NM_024773: c.482G>A (p.Cys123Tyr)
**Variant 2**		NM_024773: c.1086+14_1200_21del	NM_024773: c.1086+14_1200_21del
**Gender**		male	female
**Prenatal**		intrauterine hypotrophy, oligohydramnion, small placenta	intrauterine hypotrophy, oligohydramnion
**Birth**	weeks	35	40
	length	43 cm (- 2 SD)	43 cm (-3.5 SD)
	weight	2.36 kg (-1 SD)	2.39 kg (-2.5 SD)
	OFC	34 cm (+1 SD)	33.5 cm (-1.5 SD)
**Growth**	head	52 cm (-1.2 SD), at 10 years	51 cm (-0.5 SD), at 7 years
	weight	13.1 kg (- 4 SD)	10.2 kg (-4 SD)
	height	96.5 cm (- 7 SD)	84.1 cm (-6 SD)
**Craniofacial**	craniosynostosis	no	no
	dysmorphism	Triangular face, high forehead, broqad nasal bridge, full lips, microretrognathia, high palate, short neck, dysplastic ears	Triangular face, high forehead, deep set eyes, prominent nose, microretrognathia, short neck, dysplastic ears
**Nervous system**	Development	moderate intellectual disability	mild intellectual disability
	muscular system	muscular hypotonia	muscular hypotonia
	EEG	epileptic activity (generalised myoclonias); no treatment	Mild background abnormalities; no epileptic activity
	MRI	mild brain atrophy	mild cortical atrophy and dilated lateral ventricles
**Vascular**	cardiac	foramen ovale apertum	foramen ovale apertum, mild ventricular septal defect (closed without operation)
**Respiratory**		frequent bronchitis in infancy	frequent infections
**Skeleton**		thoracolumbar scoliosis, sacral dimple	narrow shoulders, hypoplastic nipples
**Limbs**	hands	simian crease, F5 clinodactyly	simian crease, F5 clinodactyly
	feet	right clubfeet	sandal gap
**Gastrointestinal**		reflux in infancy	No
**Genitourinary**		hypospadia, abnormal shape of right kidney (L-shape)	No
**Vision**		divergent strabismus, hyperopia	divergent strabismus, hyperopia
**Hair and nails**		No	sparse hair; mild hypoplasia of nails
**Immunological**		no	no
**Lymphatic**		no	no
**Endocrine**	IGF	43.7 μg/L	<25 μg/L (N: 55-327)
	thyroid gland	Normal	hypothyreosis
	growth hormone replacement	since 2y 2m, positive effect	since 4y, positive effect
**Other**		hematoma subduralis at 1m of age (trauma)	No
**Investigations done**		karyotype, UPD 7 and 14, methylation test for 11p15.5 abnormalities, chromosomal microarray, exome sequencing	

Supplemental Figure 1 | Table summarising clinical information related to two siblings subsequently identified as carrying biallelic *JMJD5* pathogenic variants.

Supplemental Figure 2

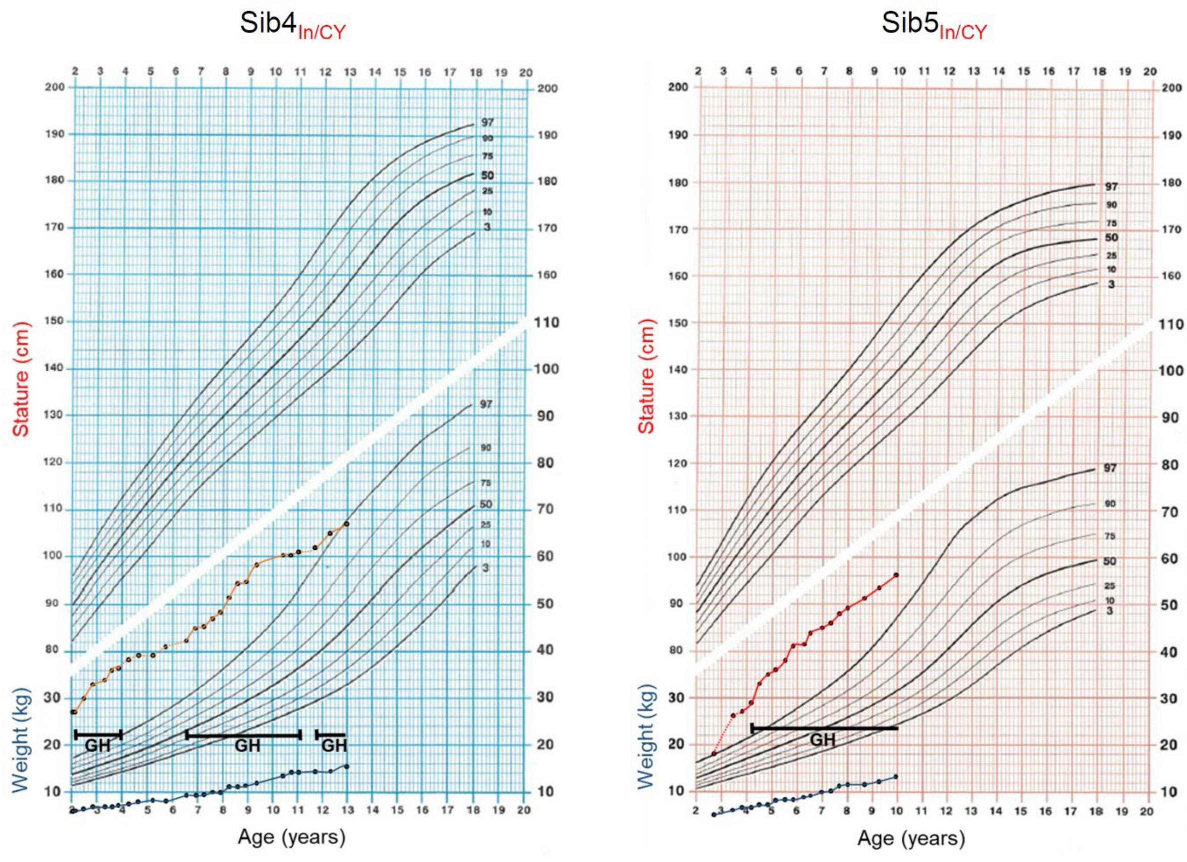

Supplemental Figure 2 | Growth charts recording the stature and weight of two siblings subsequently identified as carrying biallelic *JMJD5* pathogenic variants. Stature is recorded in red/orange (upper traces) and weight is recorded in blue (lower traces). GH = Growth Hormone. See Supplemental Patient Case Reports for a detailed description of the response of each sibling to GH therapy.

Supplemental Figure 3

ORC1	Cdc45	ATR	CUL7	DONSON	GH2
ORC2	CENPJ	ATRIP	OBSL1	XRCC4	CSHL1
ORC6	RBBP8	SMARCA4	CSH1	PCNT	CSH1
Cdt1	CEP152	SMARCB1	NF1	RNU4atac	CSH2
Cdc6	CEP63	SMARCE1	SGCE	POLE	
GMNN	NIN	ARID1A	ZFP57	GHRH	
MCM5	DNA2	ARID1B	LIG4	GH1	

Meier–Gorlin syndrome

Seckel syndrome

Coffin-Siris syndrome

Silver-Russell syndrome

Microcephalic Dwarfism

Growth Hormone Signaling

Supplemental Figure 3 | Table of genes associated with short stature and related syndromes confirmed as unaffected. Analysis of the patient WGS did not identify any variants in these genes that segregated with the clinical phenotype, thus excluding them as candidates involved in the novel developmental disorder identified here.

Supplemental Figure 4

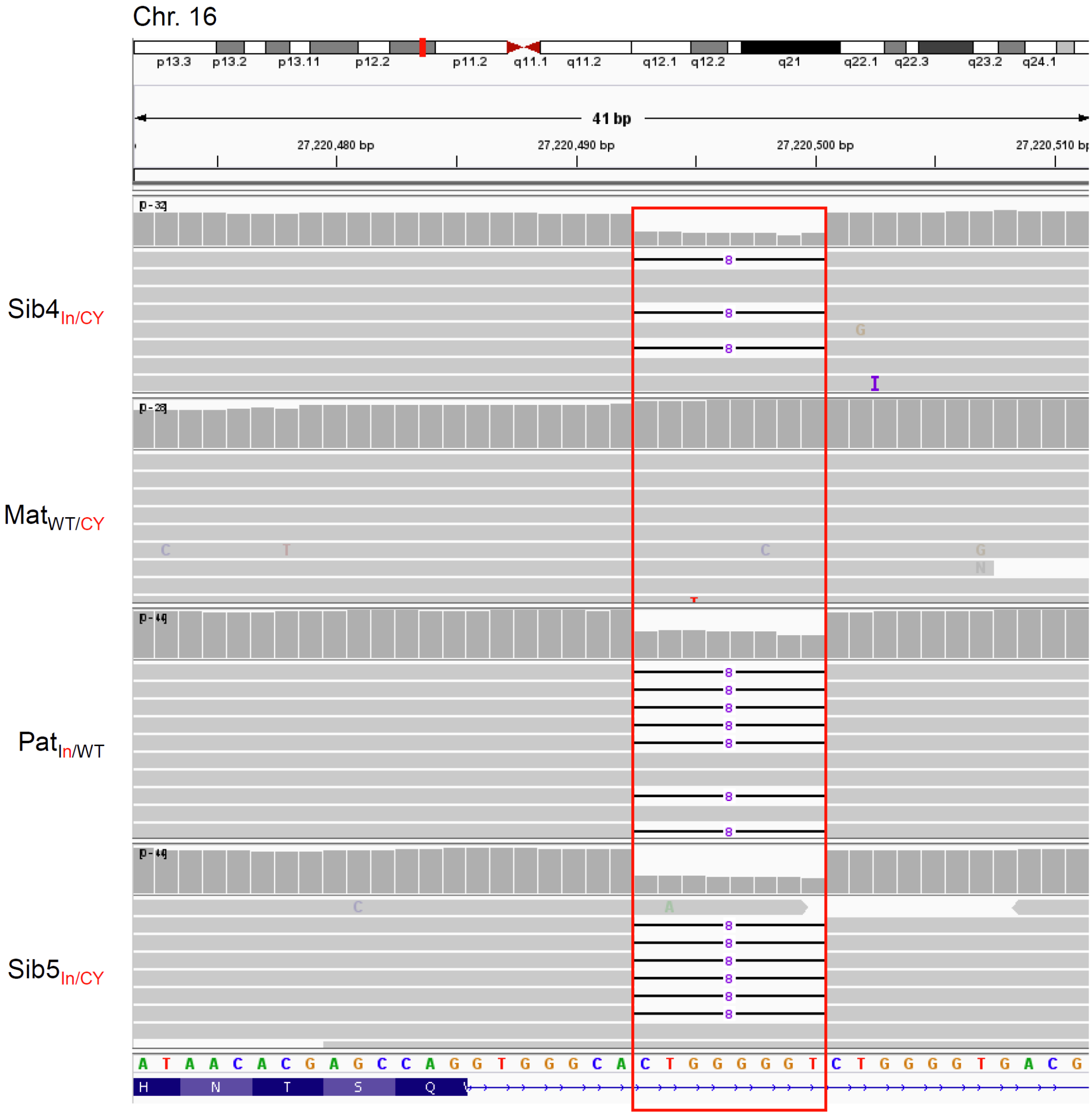

Supplemental Figure 4 | Identification of the *JMJD5* Intronic variant. WGS data was analyzed by the Integrative Genomics Viewer. Presented is a screen capture showing the intronic microdeletion present in the *JMJD5* gene on chromosome 16 of the Father (Pat) and two affected siblings (Sib). The red box highlights the deleted region.

Supplemental Figure 5

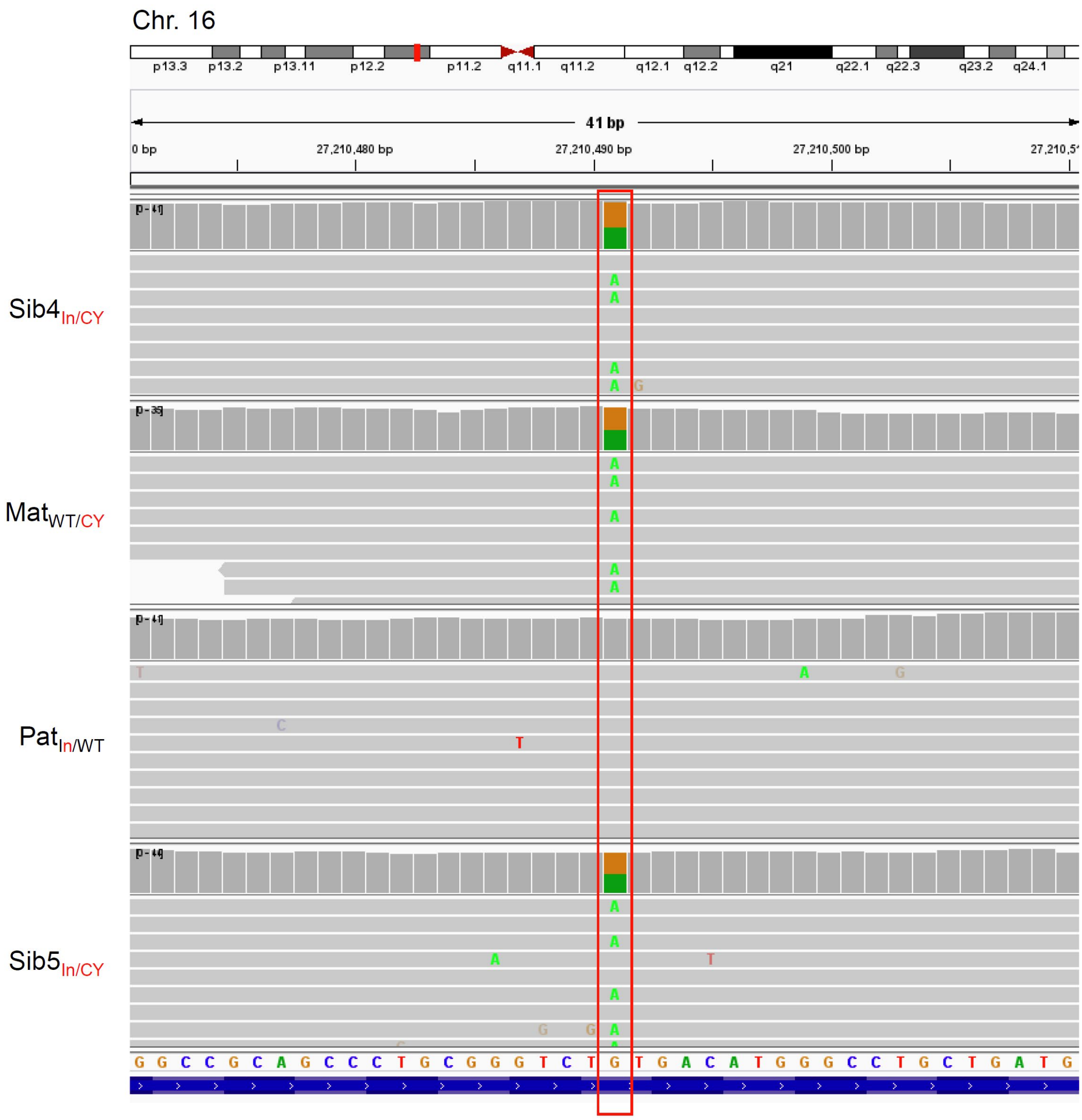

Supplemental Figure 5 | Identification of the *JMJD5* C123Y variant. WGS data was analyzed by the Integrative Genomics Viewer. Presented is a screen capture showing the C123Y missense variant present in the *JMJD5* gene on chromosome 16 of the Mother (Mat) and two affected siblings (Sib). The green text and red box highlights the single nucleotide substitution (A/G).

Supplemental Figure 6

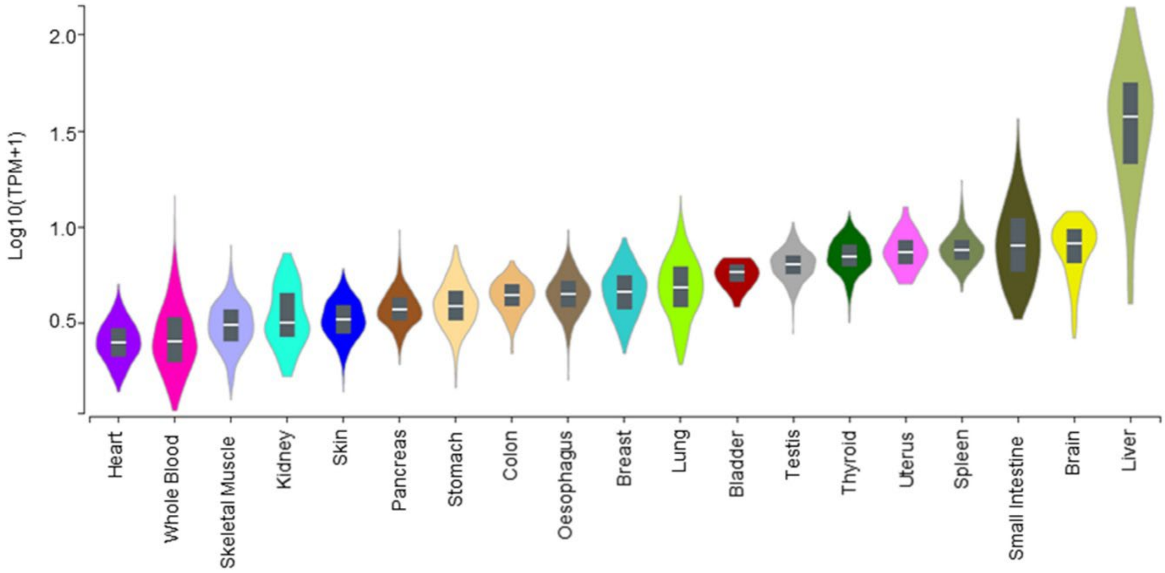

Supplemental Figure 6 | JMJD5 is expressed ubiquitously across different tissues. The online database GTEX Portal (version 8) was used to determine the expression of the human *JMJD5* gene across different tissue types. Data were filtered to display tissues from major organs only, ranked in order of increasing JMJD5 mRNA expression, and presented on a log scale. TPM units are transcripts per million normalized to gene length.

Supplemental Figure 7

A

Splice site type	Motif	Splice site	Consensus value (0-100)	
			WT	InMUT
Donor	CAGGTGGGC	CAGgtgggc	88.33	88.33
Acceptor	TGCTTTCTTCAGGT	tgctttcttcagGT	93.94	93.94
Branch Point	TCCCCAC	-	89.44	89.44
Splice Enhancer	GGGGTC	-	62.89	-

B

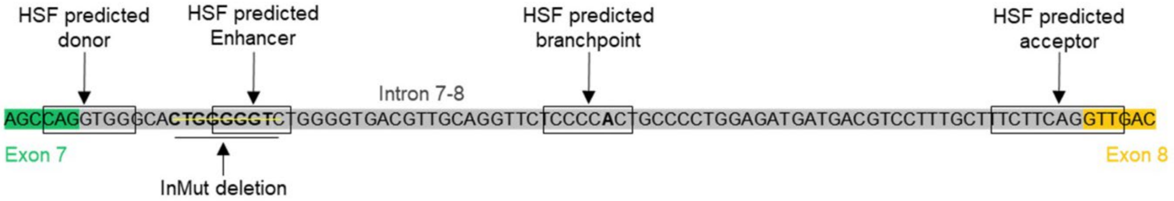

Supplemental Figure 7 | The intron deletion variant is predicted to affect JMJD5 splicing. Human Splice Finder (HSF) online software tool was used to predict splice sites for wildtype and intronic variant JMJD5 genomic DNA sequences proximal to the intron-exon boundaries of exons 7 and 8. For each potential motif the splice site was predicted and a consensus value generated; the higher the consensus value the more likely that the sequence has functional significance. (**A**) HSF analysis predicted that the highest scoring, and therefore most probable, donor and acceptor sequences (including branch point) would be unchanged between wildtype and intronic *JMJD5* genomic DNA sequences. HSF predicted the loss of an enhancer due to the Intronic variant (the sequence deleted in the enhancer is highlighted in red). (**B**) The HSF predicted splice sites and two potential splice enhancer sequences annotated onto the *JMJD5* genomic DNA sequence. HSF predicted that loss of an enhancer, due to the intronic deletion, could cause alternative splicing of the intronic variant allele.

Supplemental Figure 8

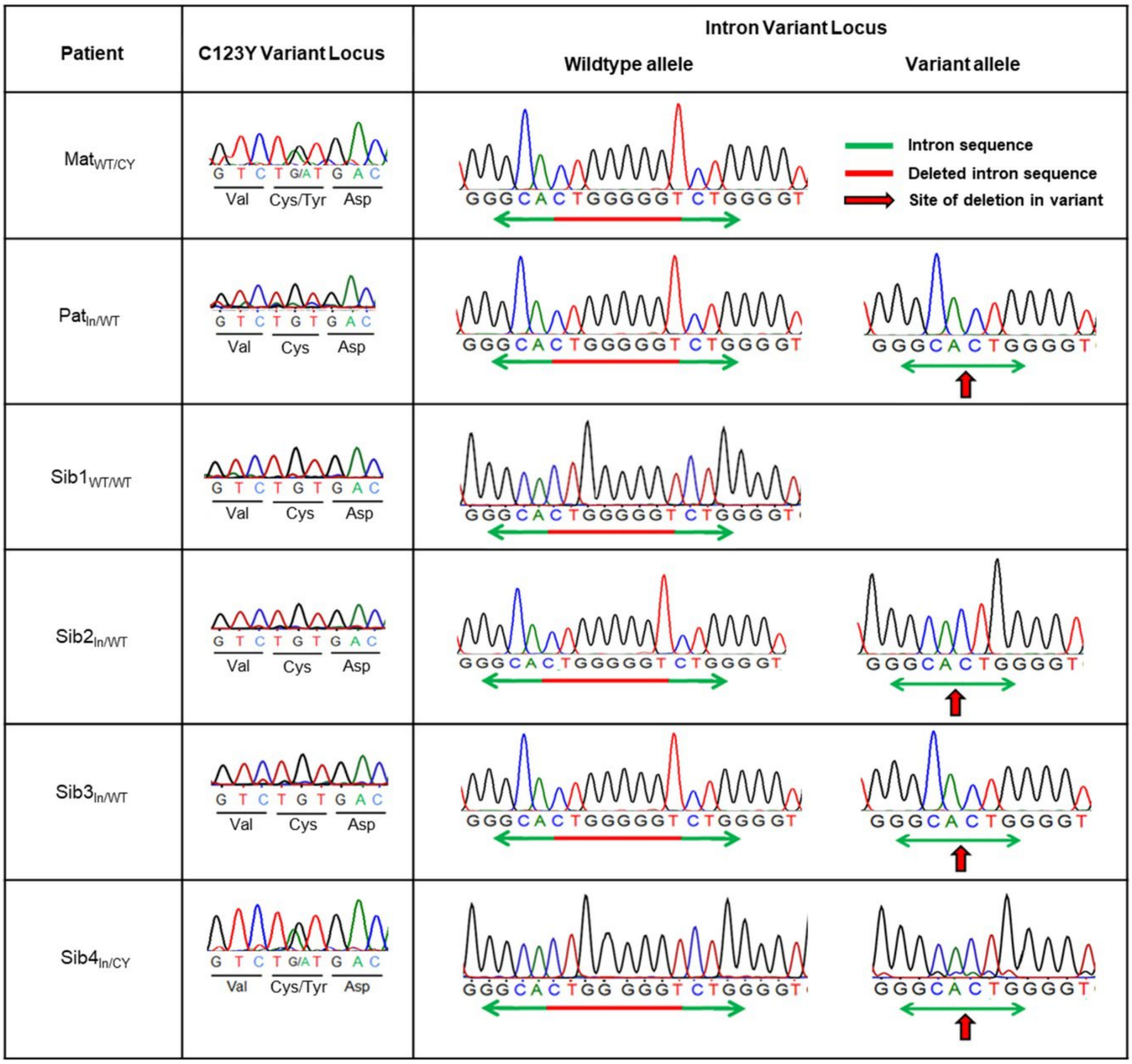

Supplemental Figure 8 | Confirming the *JMJD5* genotype of immortalized fibroblast cell lines. Genomic DNA was purified from immortalized cell lines and PCR performed to amplify the region spanning C123Y or the intronic variant. Sanger sequencing for the C123Y PCR products confirmed the presence of the C123 point variant in immortalized fibroblasts from carrier individuals ($Mat_{WT/CY}$ and $Sib4_{In/CY}$). PCR products from intronic variant samples were first TOPO-cloned then colonies screened by Sanger sequencing. This confirmed that only immortalized fibroblasts from carriers of the intronic deletion carried this variant ($Pat_{In/WT}$, $Sib2_{In/WT}$, $Sib3_{In/WT}$, $Sib4_{In/CY}$).

Supplemental Figure 9

A

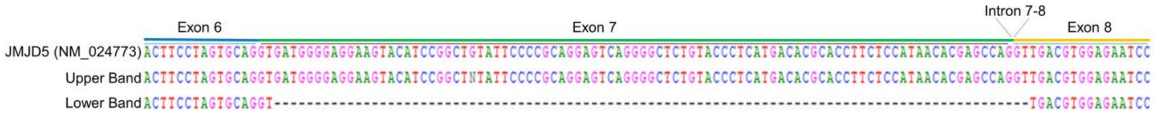

B

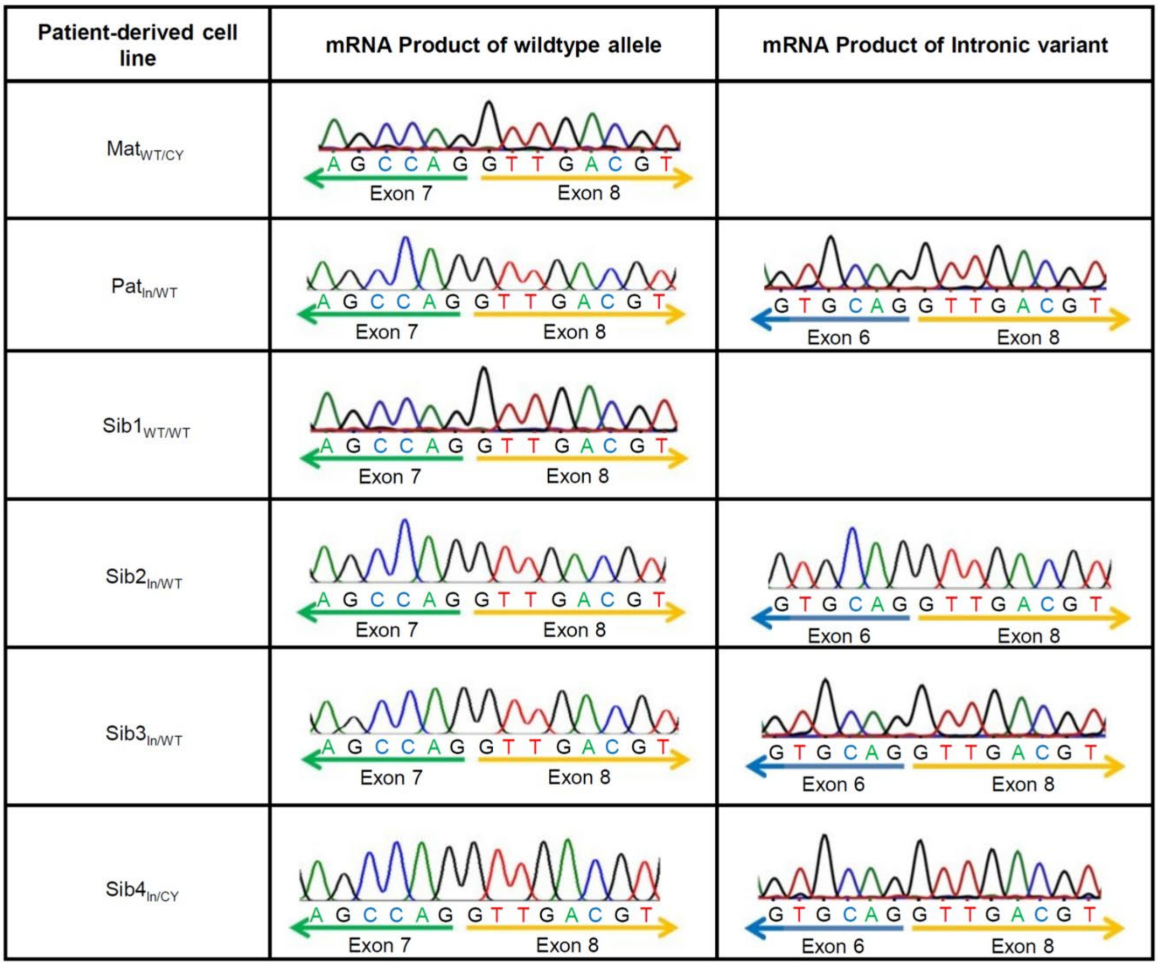

Supplemental Figure 9 | Identifying the consequences of the intronic variant on JMJD5 mRNA splicing. (**A**) RNA was purified from immortalized fibroblast lines for cDNA synthesis prior to PCR amplification of JMJD5 cDNA. Sanger sequencing of the upper and lower PCR product bands (Figure 2) and alignment to reference databases identified the 'upper band' as wildtype JMJD5 (NM_024773). The 'lower band', corresponding to the intronic variant allele, lacked the sequence encoding JMJD5 exon 7. The remainder of the sequence was normal (data not shown). (**B**) Sanger sequencing was performed on 'lower band' PCR products, as above, for all fibroblast cell lines. The removal of *JMJD5* exon 7 coding sequence was consistent for all carriers of the intronic variant. Only the Sanger sequencing across the relevant exon boundaries is shown.

Supplemental Figure 10

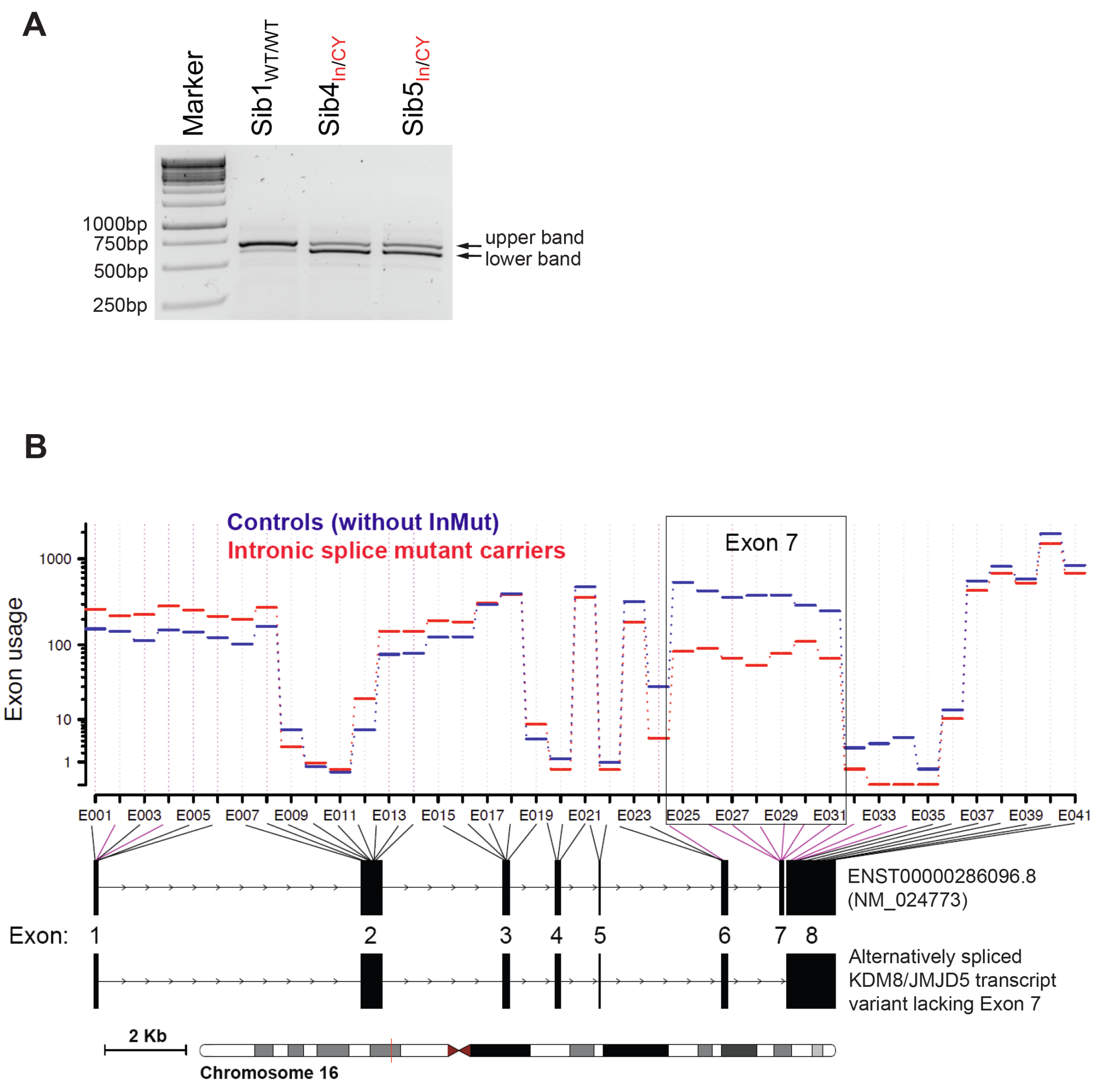

Supplemental Figure 10 | Analyses of exon usage in patient-derived cells. (**A**) An identical pattern of splicing is observed in primary patient-derived cells as immortalized patient-derived cells (see Figure 2A). JMJD5 cDNA was amplified by PCR from Exon 3 to the 3'UTR. The 'upper band' corresponds to the canonical product of 706 bp from the wildtype and C123Y allele. The 'lower band' corresponds to the alternatively spliced transcript that is enhanced by the Intronic variant. (**B**) Independent unbiased exon usage analysis of RNA-seq data obtained from immortalized cells confirms reduced exon 7 usage in individuals carrying the Intronic splice variant. Differential exon usage plots for the RNA-seq data for controls (blue traces; wildtype and C123Y carriers) and carriers of the Intronic splice variant (red traces) were analysed using DEXSeq (v 1.36). Exon usage is shown for each exon or splice junction in the KDM8 gene (Ensembl gene ENSG00000155666). The canonical Ensembl transcript (ENST00000286096.8, NM_024773) and alternatively spliced transcript are shown beneath the exon usage panel, indicating the location and organization of the corresponding numbered exonic regions (E) in the gene.

Supplemental Figure 11

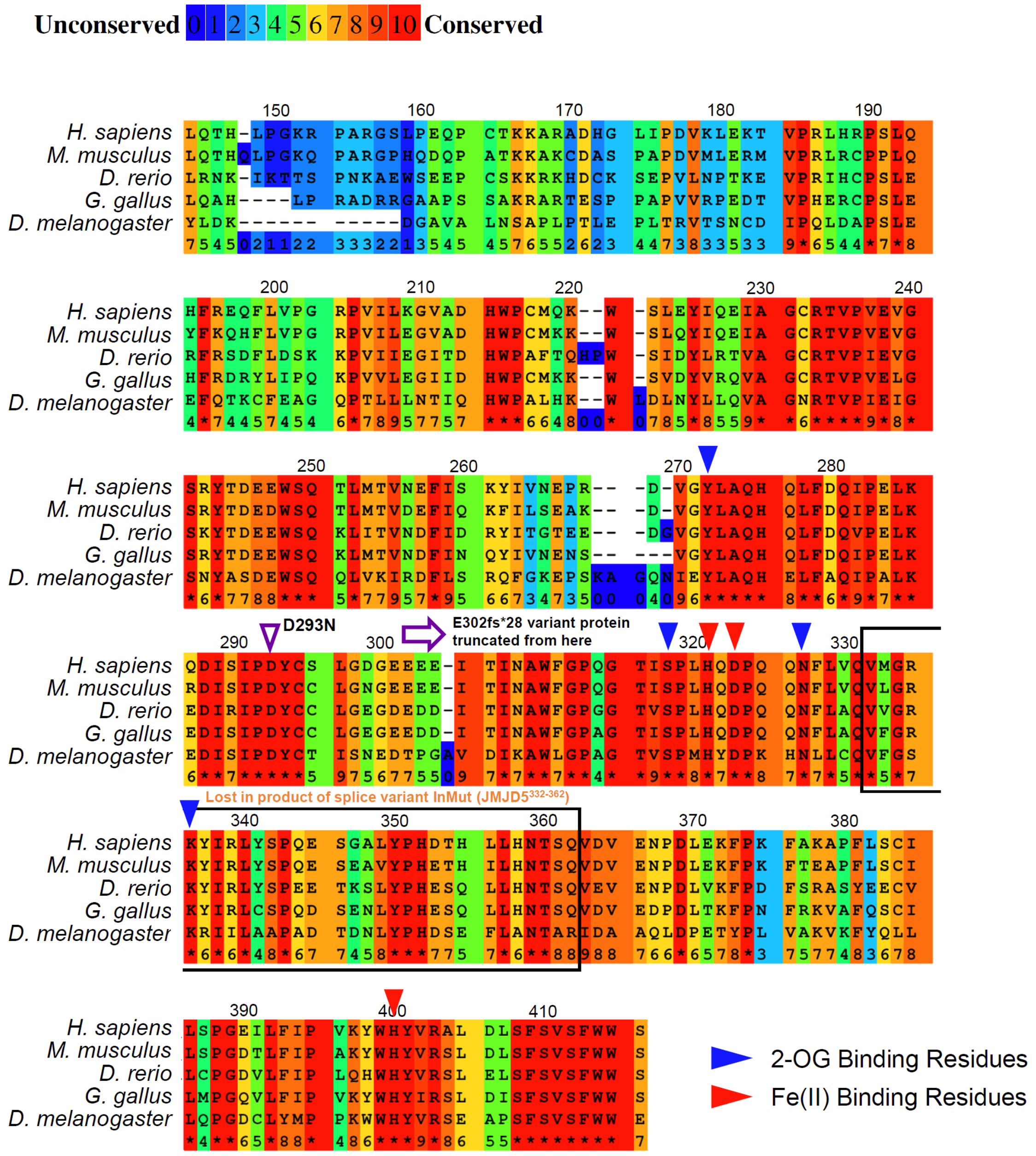

Supplemental Figure 11 | Amino acid sequence conservation of the JMJD5 catalytic domain and its critical catalytic residues. The PRALINE server was used to align the indicated species using the following protein sequences; Q8N371 (*Homo sapiens*), Q9CXT6 (*Mus musculus*), F1NW34 (*Gallus gallus*), A0A0R4IGD4 (*Danio rerio*) and M9PDS1 (*Drosophila melanogaster*). The JMJD5 catalytic domain is formed by amino acids 183 to 416 in humans. The region lost as a result of the Intronic variant is boxed in black and labelled with orange text. The position of the missense (D293N) and frameshift variants in Family 2 are shown with purple arrows (see Supplementary Figures 24-26 for more information).

Supplemental Figure 12

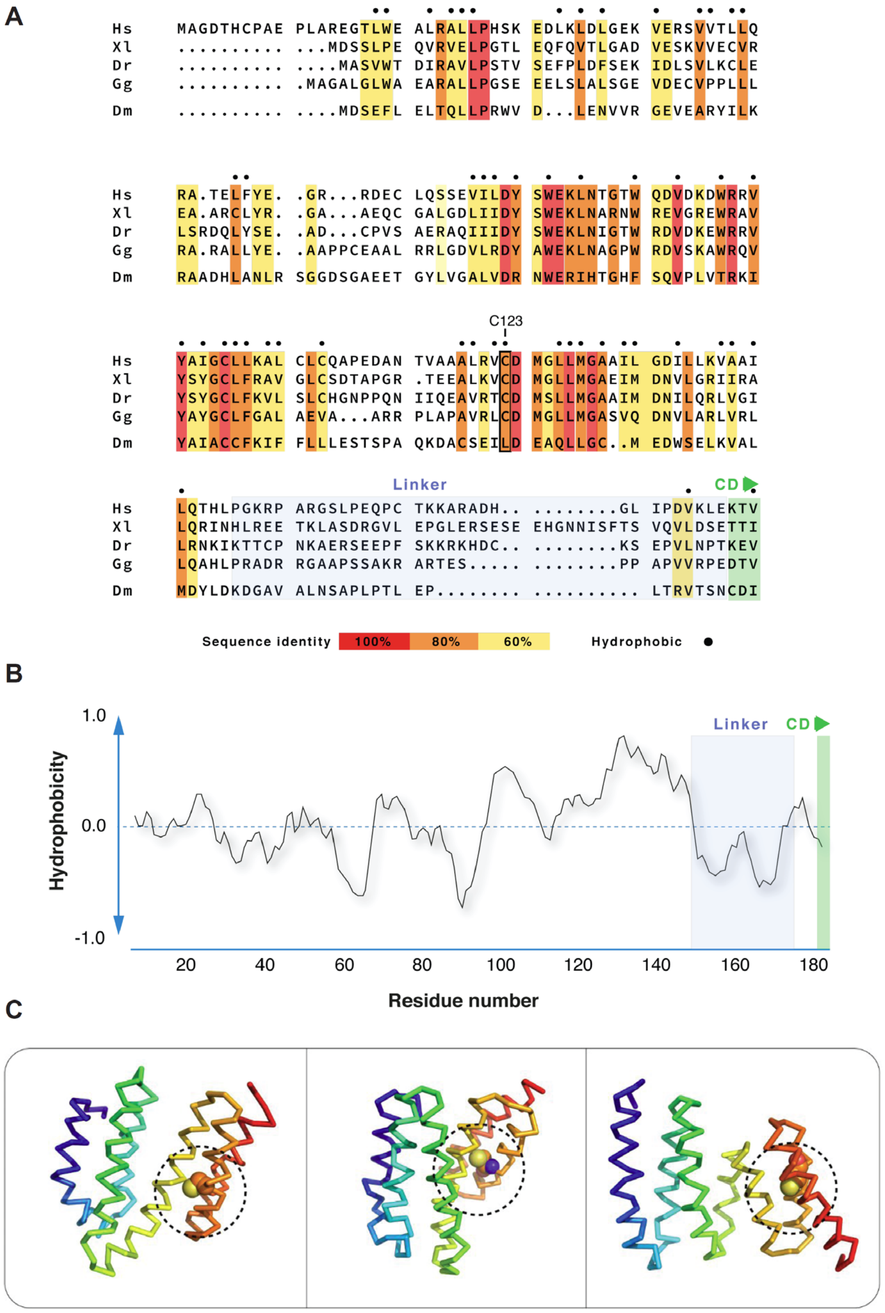

Supplemental Figure 12 | Structural characterization of the JMJD5 N-terminal region. (**A**) PRALINE alignment of the N-terminal regions of five JMJD5 orthologs (*Homo sapiens* - Hs; *Xenopus laevis* - Xl; *Danio rerio* - Dr; *Gallus gallus* - Gg; *Drosophila melanogaster* - Dm) with degree of sequence identity color coded and sequence positions conserved. Hydrophobic amino acids denoted by black dots. Variable linker region and N-terminal end of the catalytic domain (CD) are highlighted in blue and green respectively. (**B**) Hydrophobicity plot for N-terminal region. Alternating hydrophilic (negative) and hydrophobic (positive) segments (values calculated using the Eisenberg normalised consensus hydrophobicity scale and a nine residue window - https://web.expasy.org/protscale/) suggest the presence of a significant tertiary structure. Again, linker and N-terminal end of the CD are highlighted. (**C**) Cartoons of three highest scoring models derived by tertiary structure prediction for the JMJD5 N-terminal region using LOMETS (9) iterative threading as implemented in i-TASSER (10). Structures are colored with a blue-red ramp from the N- to C-termini. Divergence between models and low/intermediate C-scores reflect lack of significant homology to known structures. Nonetheless, predictions consistently suggest a helical stack architecture with C123 (space-filling representation and highlighted by dashed circles) located at hydrophobic interfaces between adjacent helical motifs.

Supplemental Figure 13

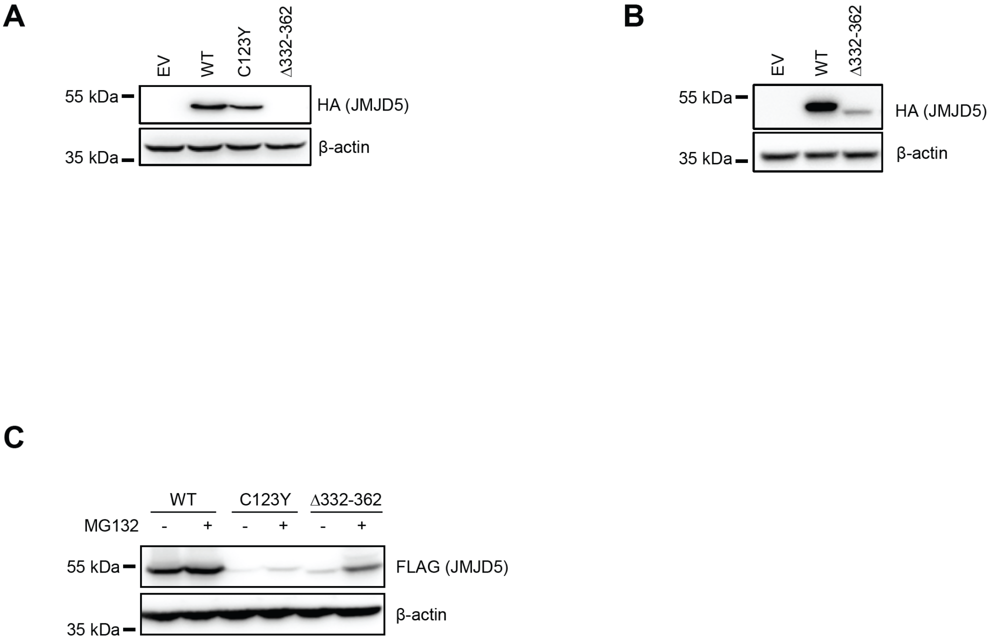

Supplemental Figure 13 | C123Y and JMJD5$^{\Delta 332\text{-}362}$ reduce the protein expression of JMJD5. (**A**) Western blotting following transient transfection of control empty vector (EV) or HA-tagged wildtype (WT), C123Y, or JMJD5$^{\Delta 332\text{-}362}$ cDNAs into HEK293T cells. (**B**) JMJD5$^{\Delta 332\text{-}362}$ migrates at a smaller molecular weight than the wildtype protein. Western blotting following transient transfection of control empty vector (EV) or HA-tagged wildtype (WT) or JMJD5$^{\Delta 332\text{-}362}$ cDNAs into HEK293T cells. A long exposure was required in order to detect the JMJD5$^{\Delta 332\text{-}362}$ variant. (**C**) The instability of JMJD5$^{\Delta 332\text{-}362}$ is proteasome-dependent. Sib1$_{WT/WT}$ cells expressing doxcycline-inducible FLAG-tagged wildtype (WT) JMJD5 (as described in Figure 3B) were treated with 10 ng/mL doxycycline for 16 h. Sib1$_{WT/WT}$ cells expressing doxcycline-inducible FLAG-tagged C123Y or JMJD5$^{\Delta 332\text{-}362}$ cDNAs were treated with 1 µg/mL doxycycline for 16 h. Subsequently cells were treated with 10 µM MG132 for 4 h, where indicated. Protein samples were Western blotted with the indicated antibodies.

Supplemental Figure 14

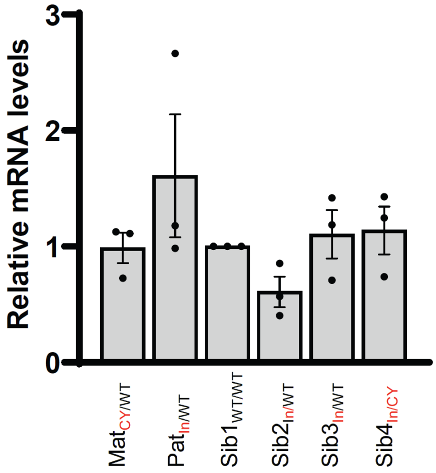

Supplemental Figure 14 | JMJD5 mRNA expression is not reduced in cells derived from affected Family 1 patients. mRNA was purified from the indicated patient-derived immortalized fibroblast lines and qPCR used to determine the level of JMJD5 mRNA expression relative to Sib1$_{WT/WT}$ cells. Data represent mean ±SEM from three independent experiments.

Supplemental Figure 15

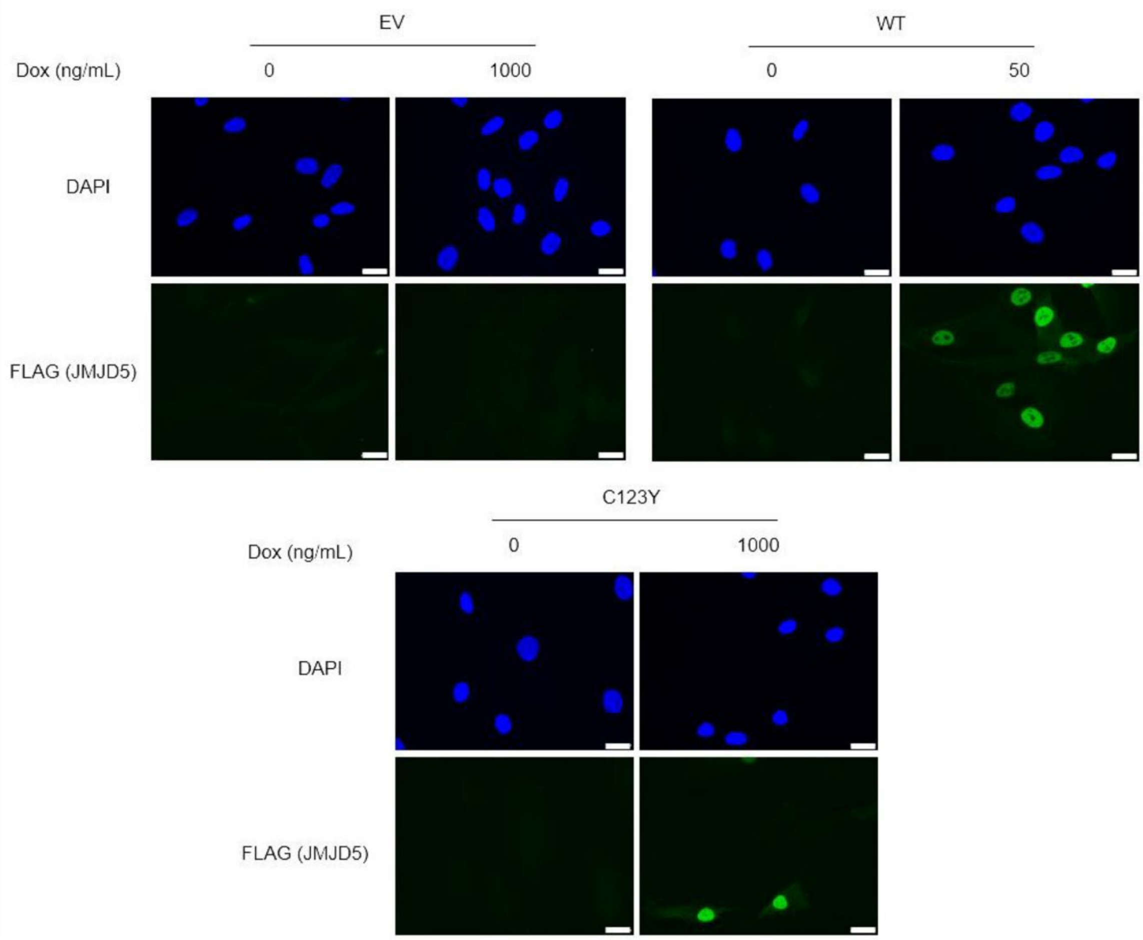

Supplemental Figure 15 | The C123Y variant does not alter the sub-cellular localization of JMJD5. Immunofluorescence was performed on Sib1$_{WT/WT}$ stable cell lines expressing doxycycline-inducible control empty vector (EV) or FLAG-tagged wildtype (WT) or C123Y JMJD5. To attempt to equalize the expression of JMJD5 in the context of the inherently unstable C123Y mutant (Figure 3), wildtype JMJD5 cells and JMJD5 C123Y cells were treated for 16 h with 50 ng/mL and 1 μg/mL doxycycline, respectively. Scale bars = 20 μm.

Supplemental Figure 16

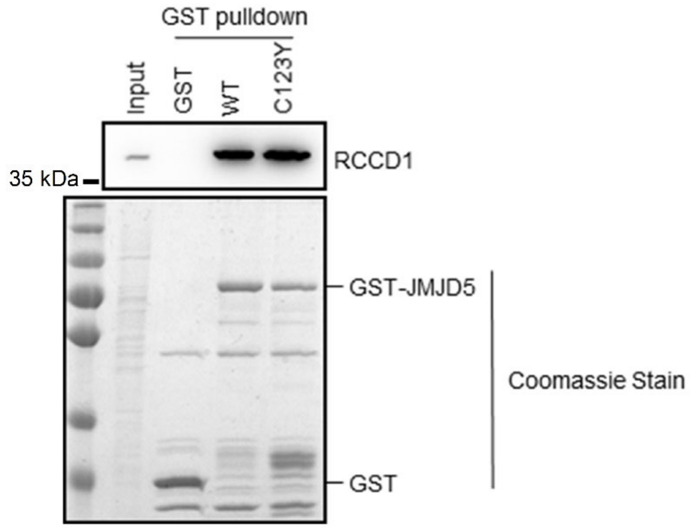

Supplemental Figure 16 | The C123Y mutation does not affect the ability of JMJD5 to interact with binding partner RCCD1. An in vitro pulldown assay was performed with GST-tagged recombinant wildtype (WT) or C123Y JMJD5 and HEK293T cell lysate expressing endogenous RCCD1, and analyzed by Western blotting (upper panel). Coomassie staining (lower panel) confirmed similar loading of GST and GST-JMJD5.

Supplemental Figure 17

A

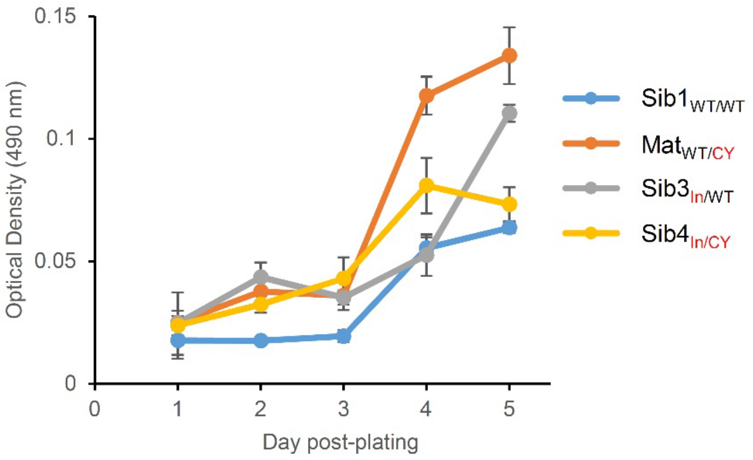

B

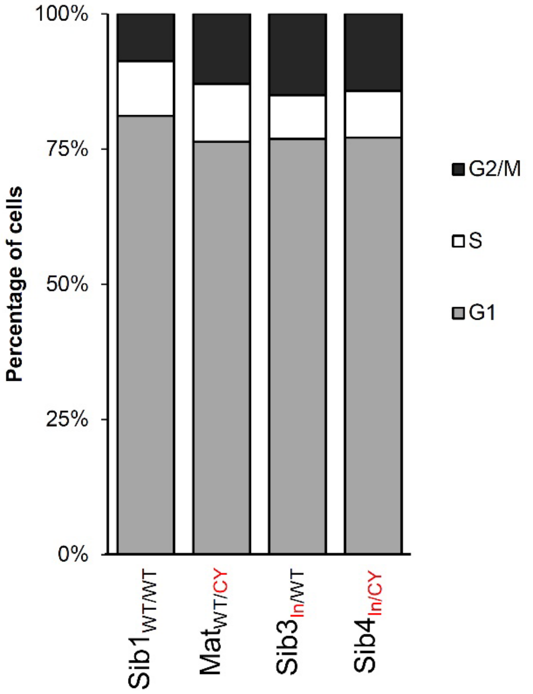

Supplemental Figure 17 | *JMJD5* genotype is not associated with differences in the growth rate or cell cycle profile of immortalized patient-derived fibroblasts. (**A**) The indicated immortalized fibroblasts were plated at low density and monitored for viable cells for five days by MTS assay. Error bars represent s.d. from three technical repeats. The experiment was repeated three times and a representative result is shown. (**B**) Cell cycle analysis of patient-derived fibroblasts using propidium iodide on a CyanB flow cytometer (Beckman Coulter). Viable cells were gated based on their forward and side scatter, then analyzed to determine cell cycle phase.

Supplemental Figure 18

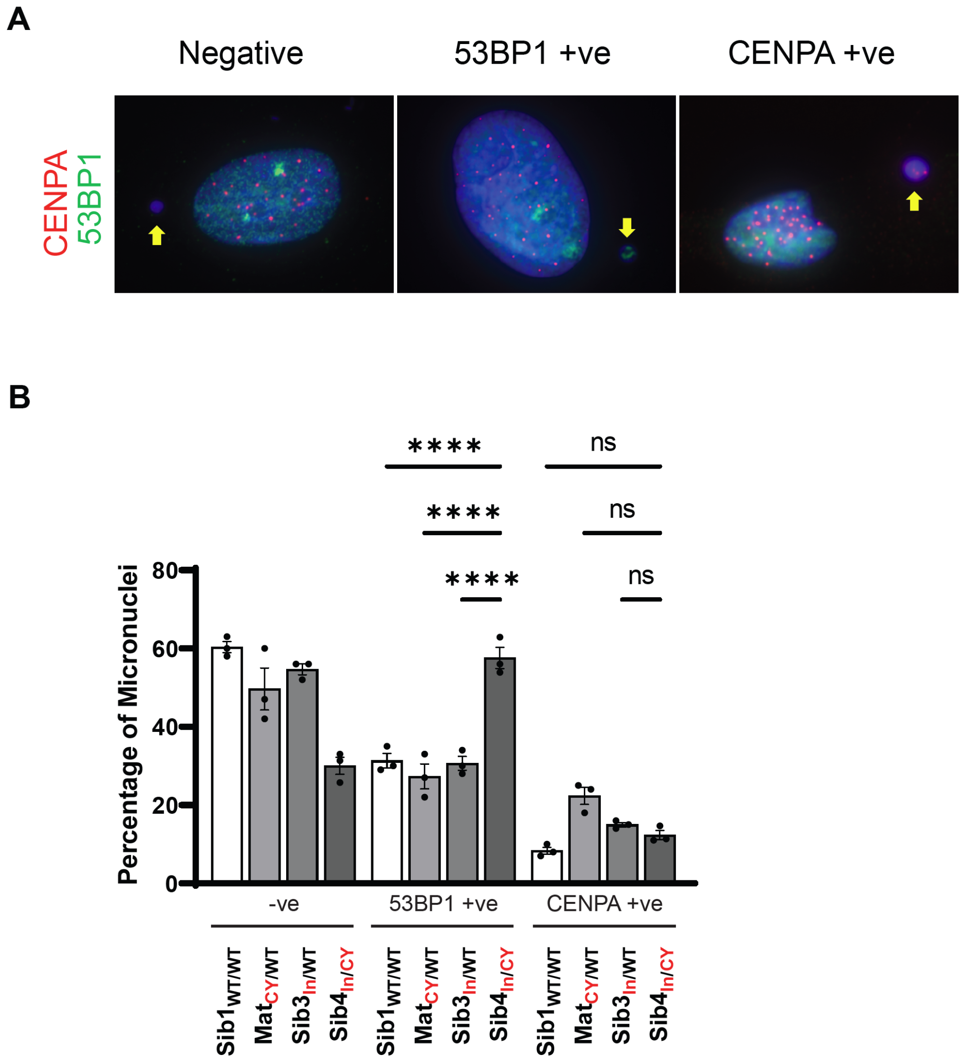

Supplemental Figure 18 | Increased spontaneous micronuclei in immortalized affected patient cells originate from unrepaired DNA damage not mitotic defects. (**A**) Representative images of DAPI-stained (blue) micronuclei (yellow arrows) co-staining with 53BP1 and CENPA. (**B**) Immortalized fibroblasts derived from the affected patient show increased 53BP1 positive micronuclei but no difference in CENPA positive micronuclei compared to unaffected cells. Data represent mean ± SEM from three independent experiments. A minimum of 100 micronuclei were counted per sample per experiment. Statistical analyses used One way ANOVA with Tukey's post hoc test with p-values of ≤0.0001 (****); ns = non-significant.

Supplemental Figure 19

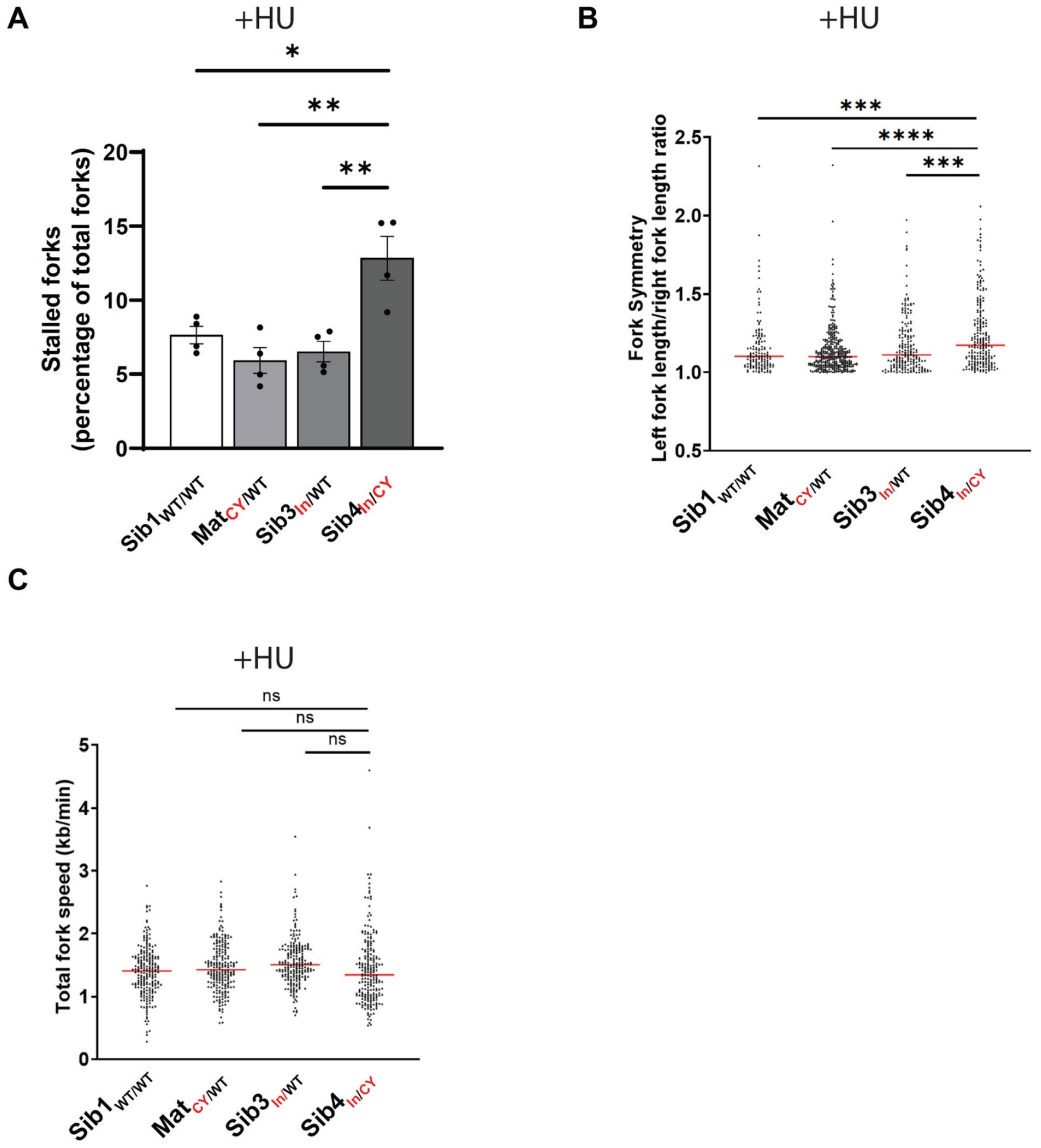

Supplemental Figure 19 | Hydroxyurea exacerbates the increased replication stress observed in cells with biallelic *JMJD5* pathogenic variants. Patient-derived fibroblasts were treated with 1 mM hydroxyurea (HU) for 2 hours between the first and second labels (see Figure 5 (B to D) for untreated cells) during the DNA fibre assay. (**A**) Stalled replication forks were increased in $Sib4_{In/CY}$ fibroblasts. A minimum of 250 structures were counted per sample in each repeat. (**B**) Asymmetric replication forks were significantly increased in $Sib4_{In/CY}$ immortalized fibroblasts. Fork symmetry was determined by measuring the ratio between the two IdU second label tracts in first label origin structures. Symmetric forks should approach a ratio of ~1.0. An increase indicates fork asymmetry. At least 50 structures were measured per sample in each experiment (**C**) Total length of at least 200 ongoing DNA fibre fork structures were measured and converted to replication fork speed. No significant difference in replication fork speed was observed. Data represent mean ± SEM from four independent experiments. Statistical analyses used One way ANOVA with Tukey's post hoc test (**A**) or Kruskal-Wallis with Dunn's correction (**B, C**), with p-values of ≤0.05 (*), ≤0.01 (**),≤0.001 (***), and ≤0.0001 (****); ns = non-significant.

Supplemental Figure 20

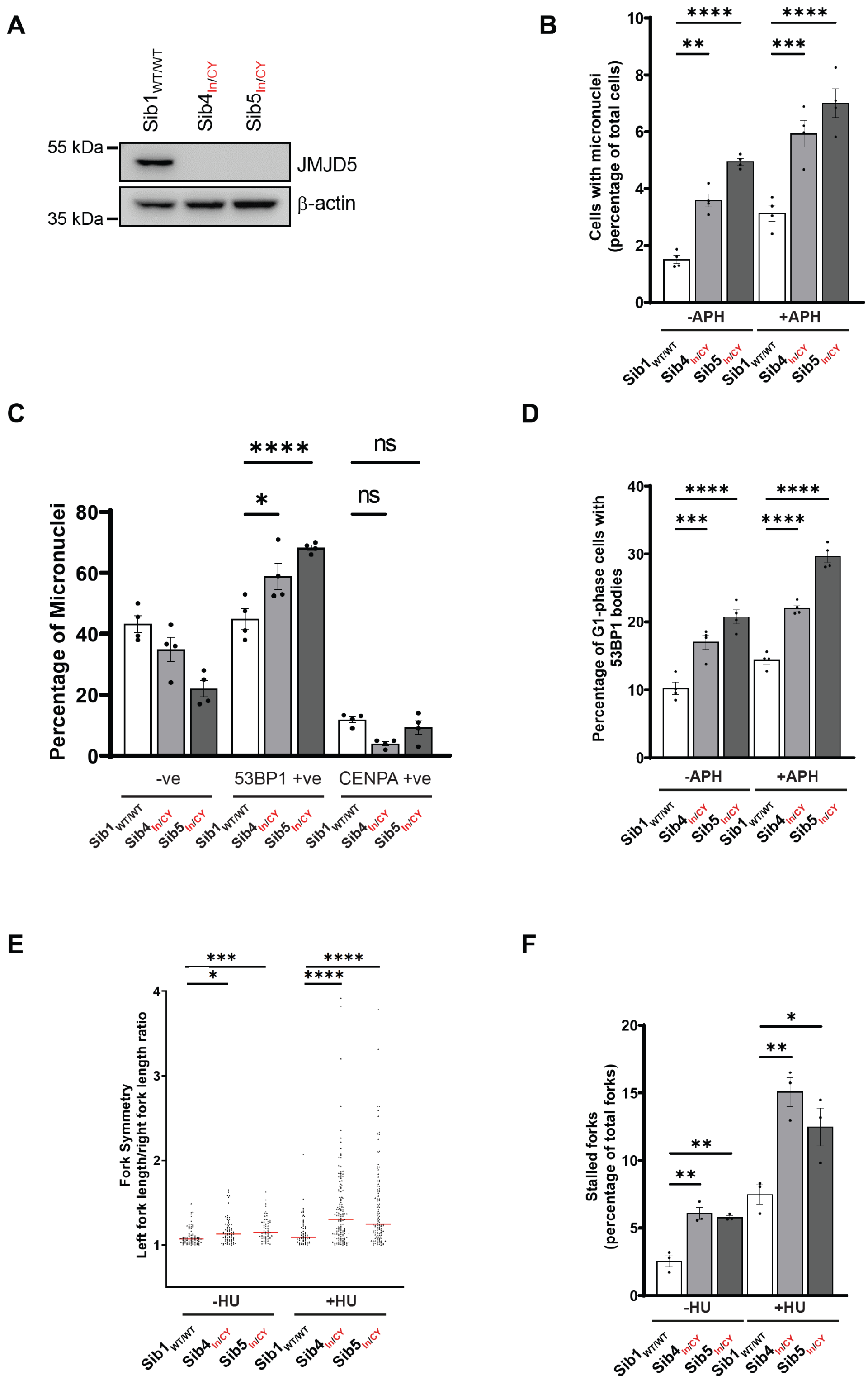

Supplemental Figure 20 | Replication stress is also associated with biallelic *JMJD5* pathogenic variants in patient-derived *primary* fibroblasts. (**A**) Western blotting of primary Sib1$_{WT/WT}$, Sib4$_{In/CY}$ and Sib5$_{In/CY}$ fibroblasts demonstrating reduced JMJD5 protein expression in cells from both affected patients (as observed in immortalized Sib4$_{In/CY}$ cells, Figure 3C). (**B**) Basal and aphidicolin (APH)-induced micronuclei are significantly higher in affected versus unaffected primary cells. In each experiment, a minimum of 500 cells were counted (**C**) Primary cells from affected patients show

increased 53BP1 positive micronuclei but no difference in CENPA positive micronuclei compared to unaffected primary cells. At least 100 micronuclei were counted per sample in each experiment. (**D**) Basal and aphidicolin (APH)-induced 53BP1 bodies in G1 cells are significantly higher in affected versus unaffected primary cells. A minimum of 300 cells were counted per sample. (**E**) Basal and Hydroxyurea (HU)-induced stalled replication forks are significantly higher in affected versus unaffected primary cells. At least 250 structures were counted per sample. (**F**) Basal and Hydroxyurea (HU)-induced replication fork asymmetry are significantly higher in affected versus unaffected primary cells. At least 50 structures were measured per sample in each experiment. (**B**-**D**) Data represent mean ± SEM from four independent experiments. (**E**, **F**) Data represent mean ± SEM from three independent experiments. Statistical analyses used One way ANOVA with Tukey's post hoc test (**B,C,D,F**) or Kruskal-Wallis with Dunn's correction (**E**), with p-values of ≤0.05 (*), ≤0.01 (**),≤0.001 (***), and ≤0.0001 (****); ns = non-significant.

Supplemental Figure 21

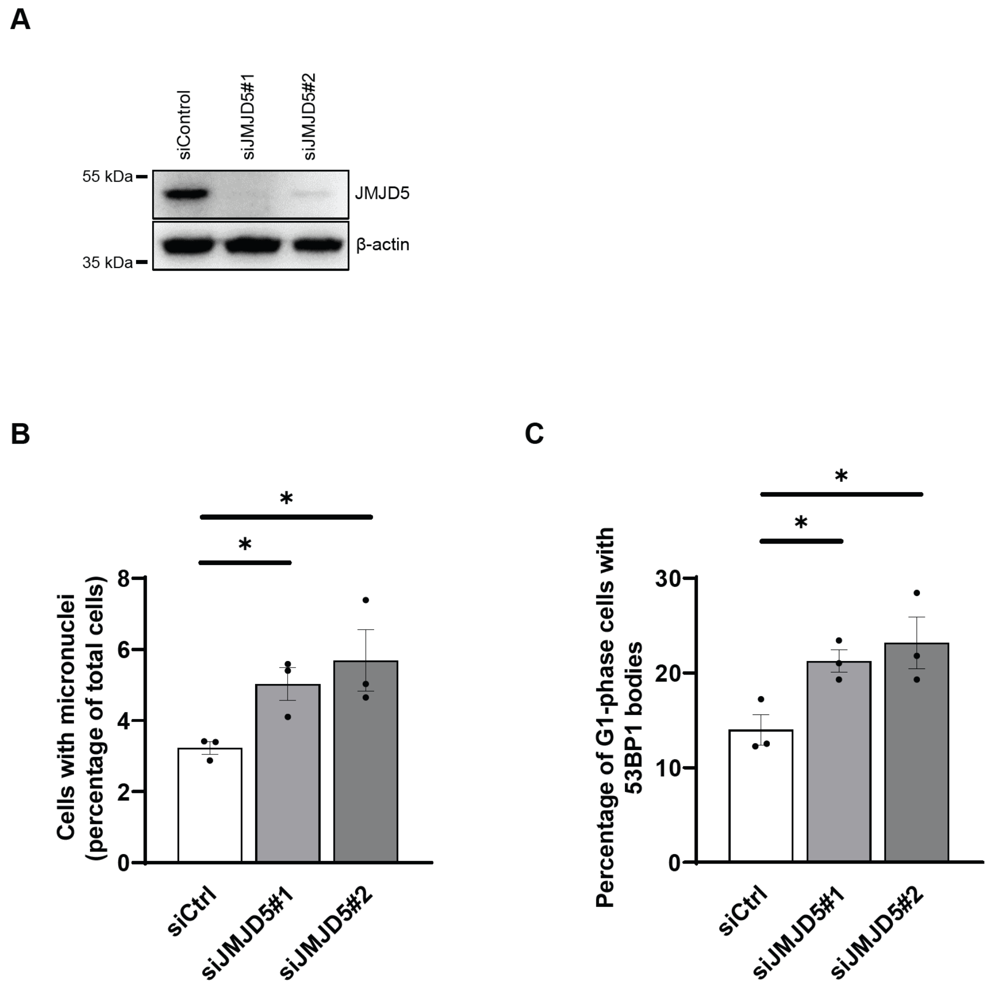

Supplemental Figure 21 | JMJD5 knockdown results in increased DNA replication stress in an immortalized epithelial cell line. The MCF10A breast epithelial cell line was transfected with control siRNA or two different siRNA sequences targeting JMJD5. (**A**) Western blotting of MCF10A cell extracts confirming successful JMJD5 knockdown. (**B**) Micronuclei were significantly increased in JMJD5 knockdown MCF10A cells. (**C**) 53BP1 bodies in G1 cells were significantly increased in JMJD5 knockdown MCF10A cells. For 53BP1 bodies, a minimum of 300 cells were counted per sample. For micronuclei, a minimum of 500 cells were counted per sample. Data represent mean ± SEM from three independent experiments. Statistical analyses used One way ANOVA with Tukey's post hoc test with p-values of ≤0.05 (*).

Supplemental Figure 22

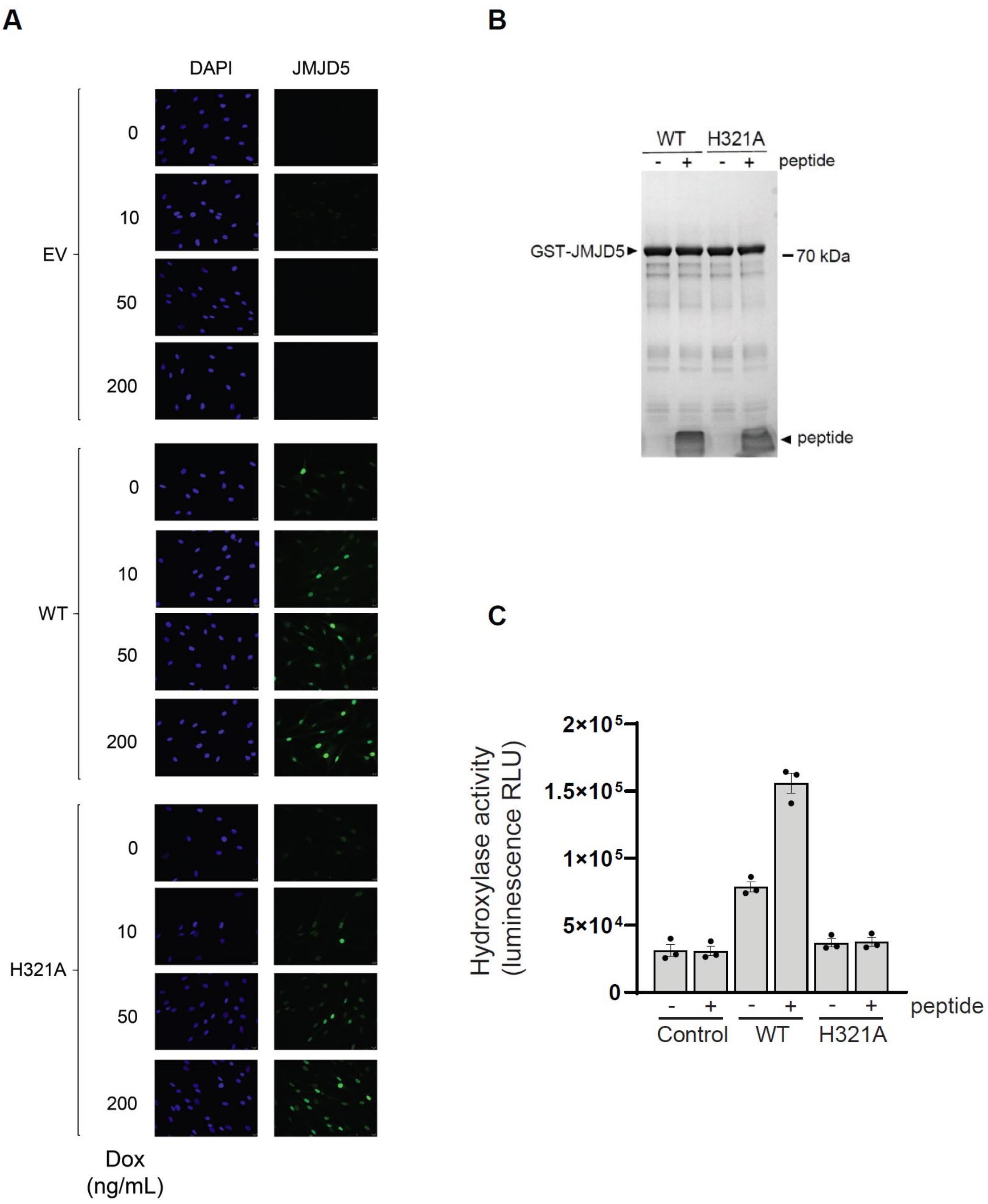

Supplemental Figure 22 | Development of a conditional JMJD5 re-expression system. (**A**) Immunofluorescence was performed on Sib4$_{In/CY}$ stable cell lines expressing doxycycline-inducible control empty vector (EV), wildtype (WT) or inactive (H321A) JMJD5 following 14 days treatment with the indicated doses of doxycycline. Note leaky expression in the absence of doxycycline and the dose-dependent increase in JMJD5 expression and nuclear localization. 0 and 10 ng/mL doxycycline doses were selected for functional experiments. See Figure 6A for Western blot validation. (**B**) and (**C**) Purified recombinant JMJD5 H321A mutant lacks hydroxylase activity in vitro. GST-tagged wildtype (WT) and H321A JMJD5 proteins were expressed in *E.coli* and purified by glutathione chromatography prior to in vitro enzyme assays with a synthetic substrate peptide. (B) Coomassie stained SDS-PAGE gel of assay samples. (C) Activity was monitored using the succinate-Glo assay, which measures succinate production. Data represent mean ± SEM from three independent experiments.

Supplemental Figure 23

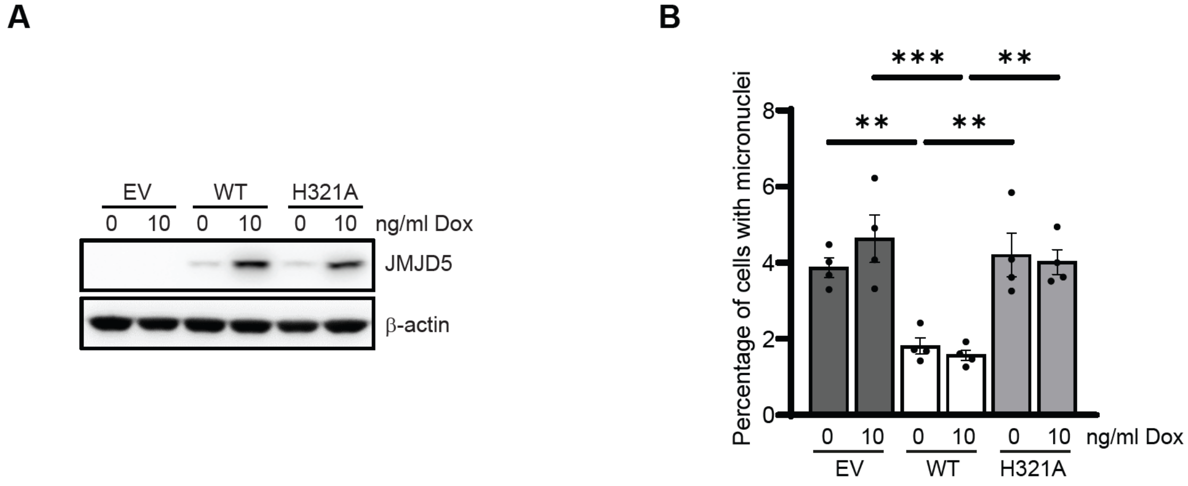

Supplemental Figure 23 | Increased replication stress in affected patient cells is due to loss of JMJD5 hydroxylase activity Sib4$_{In/CY}$ cells with stable expression of doxycycline-inducible control empty vector (EV), wildtype (WT) or inactive (H321A) JMJD5 were treated with the indicated doses of doxycycline for 14 days prior to analysis. **(A)** Western blot showing exogenous JMJD5 expression (see also Figure 6A and Supplemental Figure 22A). **(B)** Increased micronuclei in affected patient cells is due to loss of JMJD5 hydroxylase activity. Data represent mean ± SEM from four independent experiments. Statistical analyses used One way ANOVA with Tukey's post hoc test with p-values of ≤0.05 (*), ≤0.01 (**) and ≤0.001 (***). A minimum of 500 cells were counted per sample.

Supplemental Figure 24

A

Patient		Family 2 - Sib1 $_{E302fs*28/D293N}$
**Primary clinician**		Rachel Rabin/John Pappas, New York
**Gene**		*KDM8/JMJD5*
**Variant 1**		NM_024773: c.877 G>A p.(D293N)
**Variant 2**		NM_024773: c905del p.(E302Gfs*28)
**Gender**		Male
**Prenatal**		pre-eclampsia, severe intra-uterine growth retardation
**Birth**	weeks	27 weeks and 6 days
	weight	770 grams
**Growth**	head	46.5 cm at 32 months
	weight	9.6 kg at 3 years
	height	84.1 cm at 3 years
**Craniofacial**	craniosynostosis	no
	dysmorphism	Progeric appearance with periorbital fullness
**Nervous system**	Development	non-verbal at 3 years
	muscular system	muscular hypotonia
	EEG	No EEG; history of one simple febrile seizure at 2y4m
**Respiratory**		chronic lung disease due to prematurity; intubated for about 1 month after birth
**Gastrointestinal**		G-tube placed in neonatal intensive care unit and removed around 15 months. Vomiting and oral aversion
**Genitourinary**		bilateral inguinal hernia repair
**Vision**		myopia
**Hair and nails**		hair thinning
**Investigations done**		Microarray showed de novo duplication at 15q21.3(54888930_55744654)

B

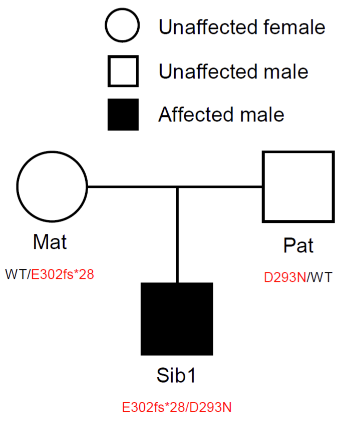

Supplemental Figure 24 | (A) Table summarising clinical information related to the affected child of Family 2 subsequently identified as carrying biallelic *JMJD5* pathogenic variants. **(B)** Pedigree of Family 2. The *JMJD5* genotype is annotated for each individual ('Mat' = maternal, 'Pat' = paternal, 'Sib' = sibling).

Supplemental Figure 25

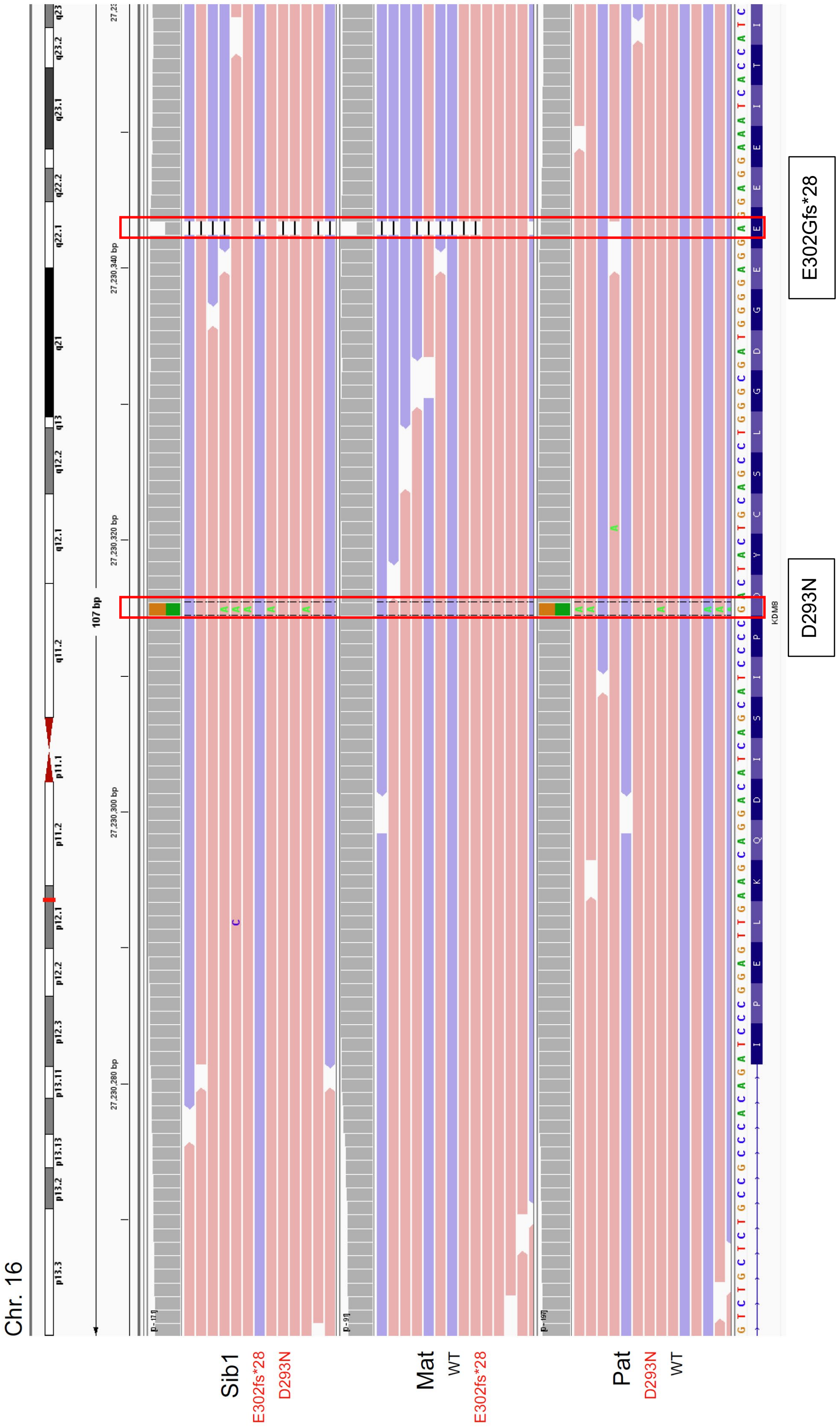

Supplemental Figure 25 | Identification of the *JMJD5* variants of Family 2. WGS data was analyzed by the Integrative Genomics Viewer. Presented is a screen capture showing the missense (D293N) and frameshift (E302Gfs*28) variants present in the *JMJD5* gene on chromosome 16 of the Father (Pat), Mother (Pat) and affected child (Sib1). The red boxes highlights the variants.

Supplemental Figure 26

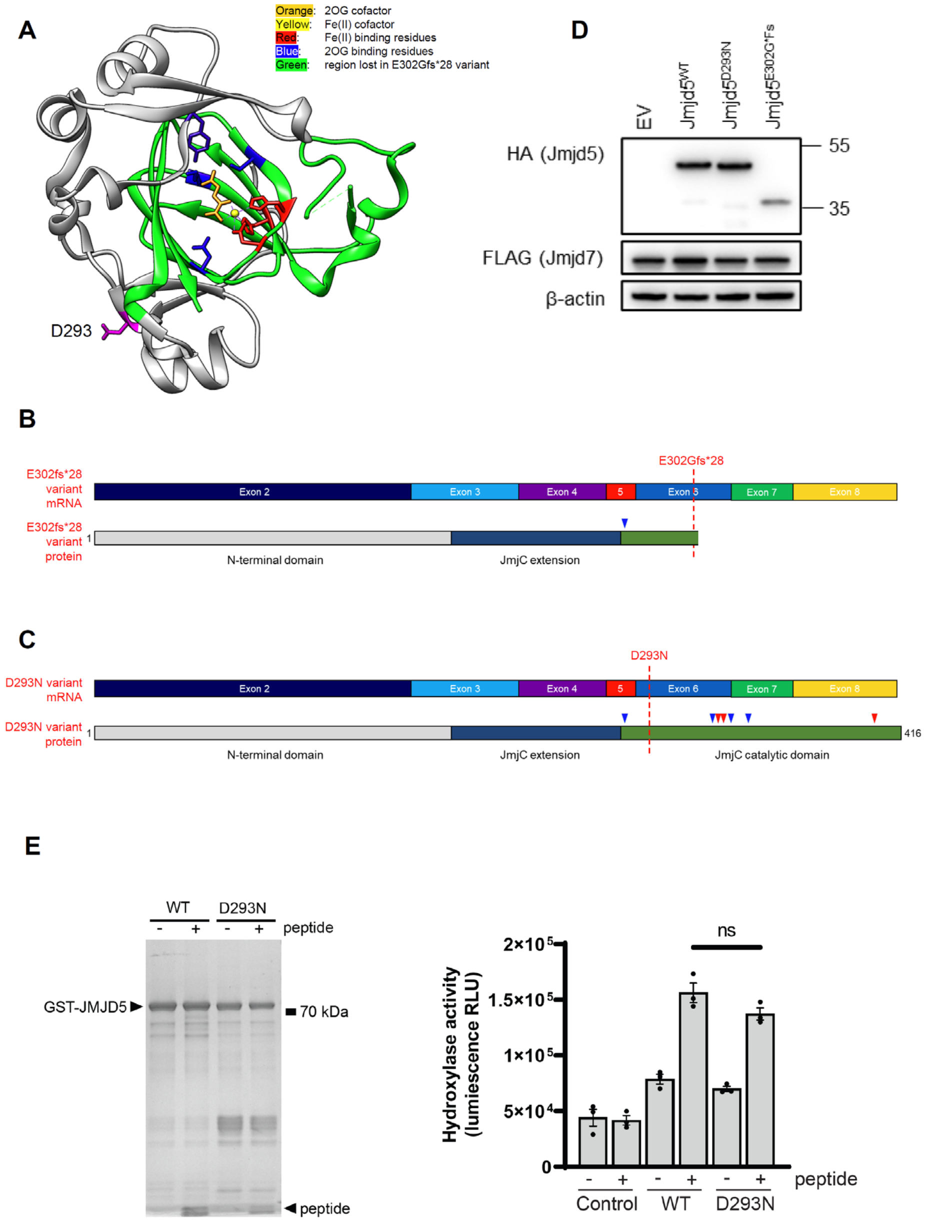

Supplemental Figure 26 | (A) Structure of catalytic domain of JMJD5 (PDB: 4GAZ) with critical catalytic residues labelled. The region missing from the JMJD5 E302Gfs*28 variant is highlighted in green. D293 is shown in purple. (**B**) Graphical representation of E302Gfs*28 frameshift variant mRNA (top) and protein (bottom) demonstrating loss of sequence integral to the catalytic domain. Fe(II)- and 2OG-binding residues are marked by red and blue arrow heads, respectively. (**C**) Graphical representation of the D293N missense variant mRNA (top) and protein (bottom). (**D**) The D293N variant reduces JMJD5 expression. Western blotting following transfection of HA-tagged JMJD5 cDNAs and transfection control (FLAG-JMJD7) into HEK293T cells. (**E**) Partially purified recombinant JMJD5 D293N retains hydroxylase activity against a synthetic peptide in vitro. GST-tagged wildtype (WT) and D293N JMJD5 were expressed in *E.coli* and purified prior to in vitro activity assays. Each reaction was also analyzed by Coomassie gel (*left*). Activity was monitored using the succinate-Glo assay, which measures succinate production (*right*). Data represent mean ± SEM from three independent experiments. Statistical analysis used One way ANOVA with Tukey's post hoc test with p-values of $p \leq 0.05$ considered significant; ns = non-significant.

Supplemental Figure 27

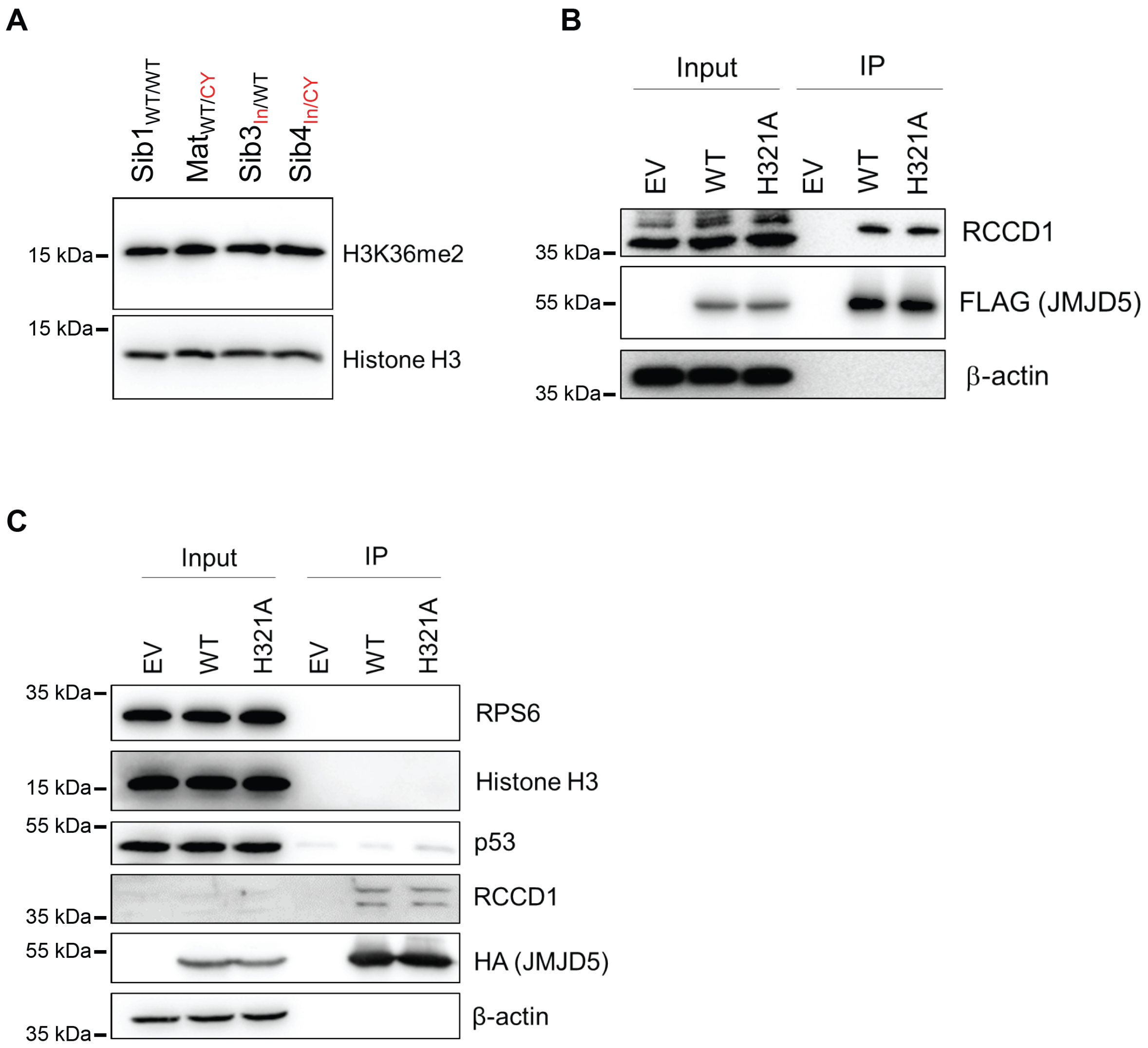

Supplemental Figure 27 | Reported JMJD5-dependent interactions and phenotypes. (**A**) Western blot with the indicated antibodies of chromatin fractions isolated from patient-derived immortalized fibroblasts. (**B**) RCCD1 interacts with JMJD5 in an activity-independent manner. Lysates from HEK293T cells transfected with control pIPZ (EV) or pIPZ 3XFLAG-JMJD5 wildtype (WT) or inactive mutant (H321A) were anti-FLAG immunoprecipitated (IP) before Western blotting with the indicated antibodies. Consistent with RCCD1 binding to the N-terminus of JMJD5 (11), its interaction with wildtype JMJD5 was similar to the hydroxylase defective JMJD5 mutant. Conversely, JmjC-hydroxylases often interact with their substrates in an activity-dependent manner (12, 13). (**C**) No specific or detectable interaction was identified between JMJD5 and reported substrates. HEK293T cells were transfected with empty vector control (EV), or HA-tagged wildtype (WT) or inactive (H321A) JMJD5 prior to anti-HA immunoprecipitation and Western blotting with the indicated antibodies.

Supplemental Figure 28

A

mRNA expression in affected vs unaffected immortalized cells

Gene	Symbol	log2FoldChange	lfcSE	padj
ENSG00000130741	EIF2S3	0.40	0.24	0.32
ENSG00000067048	DDX3Y	-0.88	2.40	0.88
ENSG00000183878	UTY	-0.27	1.54	0.95
ENSG00000012817	KDM5D	-0.96	2.12	0.85

B

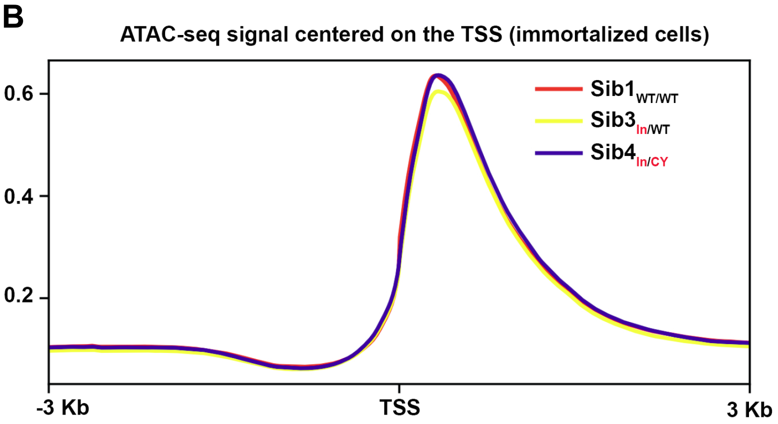

C

mRNA expression in affected vs unaffected primary cells

Gene	Symbol	log2FoldChange	lfcSE	padj
ENSG00000130741	EIF2S3	-0.44	0.17	0.07
ENSG00000067048	DDX3Y	1.16	1.41	0.69
ENSG00000183878	UTY	0.87	1.41	0.78
ENSG00000012817	KDM5D	1.19	1.44	0.69

D

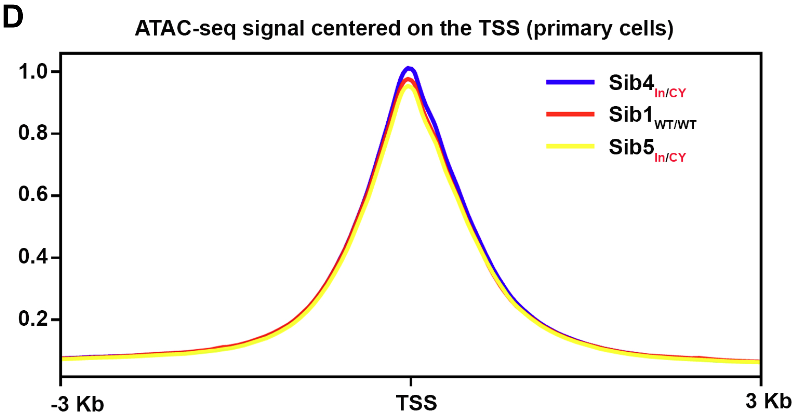

Supplemental Figure 28 | Genomic characterization of Family 1 patient-derived cells. Expression of the indicated mRNA transcripts is not significantly altered in affected versus unaffected patient-derived cells as determined by RNAseq of the indicated immortalized (**A**) and primary (**C**) cell lines (see reference(14)). lfcSE = Standard Error of the log fold change. padj = adjusted p-value. Global chromatin accessibility is not altered in cells derived from affected patients. Shown is the average ATAC-seq enrichment relative to the transcription start site (TSS) of all expressed genes plotted for the indicated immortalized (**B**) and primary (**D**) cell lines. No significant difference in accessibility was observed.

Supplemental Figure 29

A

Primer	Sequence (5' to 3')
C123Y (cDNA)	Forward: CACATGGCAGGACGTAGACAA
	Reverse: GCCTCTTTCCAGGGAGGTGT
C123Y (gDNA)	Forward: CACATGGCAGGACGTAGACAA
	Reverse: GCCTCTTTCCAGGGAGGTGT
Intronic variant (cDNA)	Forward: TCCAGCATTTCAGGGAGCAG
	Reverse: ATGTCAGGCCCTTTCAGCTC
Intronic variant (gDNA)	Forward: CTGTATTCCCCGCAGGAGTC
	Reverse: CAGGAGACAGGATGCAGGAC

B

Sequence name	Catalogue Number	Sequence 5'-3'
siJMJD5#1	SASI_Hs01_00082888	CCCUCAUGACGGUCAACGA
siJMJD5#2	SASI_Hs01_00082891	CUGACAUGCAGACAGCAUU

C

Target	Antibody
β-Actin	HRP-tagged, Abcam AB49900
JMJD5	Matsuura Yoshiharu Laboratory, Osaka University, Japan
HA	HRP-tagged Sigma-Aldrich, (12013819001)
FLAG	HRP-tagged Sigma-Aldrich, A8592
α-Goat-HRP	DAKO, P0449
α-Rabbit-HRP	Cell Signalling Technology 7074S
α-Mouse-HRP	Cell Signalling Technology 7076S
RCCD1	Santa Cruz Biotechnology sc240810
Histone H3	Abcam AB1791
p53	Calbiochem, OP43
H3K36me2	Abcam AB9049
RPS6	Cell Signalling Technology 5G10

D

Target	Antibody
FLAG	Sigma-Aldrich, F1804
CENPF	BD Biosciences, 610768
53BP1	Bio-Techne, NB100-904V
JMJD5	Custom polyclonal rabbit antibody, Bethyl Labs
BrdU (recognises CldU)	Abcam, ab6326
BrdU (recognises IdU)	BD Biosciences, 347580
Mouse 555nm	Thermofisher, A31570
Mouse 488nm	Thermofisher, A10684
Rabbit 488nm	Thermofisher, A11070
Rabbit 555nm	Thermofisher, A21430
Rat 555nm	Thermofisher, A-21434

Supplemental Figure 29 | Tables of reagents applied in this study. (**A**) Primers used for genotyping JMJD5 variants using cDNA or gDNA samples. (**B**) JMJD5 siRNA sequences. (**C**) Western blot antibodies. (**D**) Immunofluorescence antibodies.

Supplemental References

1. Wakeling EL, et al. Diagnosis and management of Silver-Russell syndrome: first international consensus statement. *Nature reviews Endocrinology.* 2017;13(2):105-24.
2. Buenrostro JD, et al. ATAC-seq: A Method for Assaying Chromatin Accessibility Genome-Wide. *Curr Protoc Mol Biol.* 2015;109:21 9 1- 9 9.
3. Corces MR, et al. Lineage-specific and single-cell chromatin accessibility charts human hematopoiesis and leukemia evolution. *Nat Genet.* 2016;48(10):1193-203.
4. Li H, and Durbin R. Fast and accurate short read alignment with Burrows-Wheeler transform. *Bioinformatics.* 2009;25(14):1754-60.
5. Ramirez F, et al. deepTools2: a next generation web server for deep-sequencing data analysis. *Nucleic acids research.* 2016;44(W1):W160-5.
6. Dobin A, and Gingeras TR. Mapping RNA-seq Reads with STAR. *Curr Protoc Bioinformatics.* 2015;51:11 4 1- 4 9.
7. Anders S, et al. HTSeq--a Python framework to work with high-throughput sequencing data. *Bioinformatics.* 2015;31(2):166-9.
8. Liao Y, et al. featureCounts: an efficient general purpose program for assigning sequence reads to genomic features. *Bioinformatics.* 2014;30(7):923-30.
9. Zheng W, et al. LOMETS2: improved meta-threading server for fold-recognition and structure-based function annotation for distant-homology proteins. *Nucleic acids research.* 2019;47(W1):W429-W36.
10. Yang J, et al. The I-TASSER Suite: protein structure and function prediction. *Nature methods.* 2015;12(1):7-8.
11. Marcon E, et al. Human-chromatin-related protein interactions identify a demethylase complex required for chromosome segregation. *Cell reports.* 2014;8(1):297-310.
12. Feng T, et al. Optimal translational termination requires C4 lysyl hydroxylation of eRF1. *Molecular cell.* 2014;53(4):645-54.
13. Markolovic S, et al. The Jumonji-C oxygenase JMJD7 catalyzes (3S)-lysyl hydroxylation of TRAFAC GTPases. *Nat Chem Biol.* 2018;14(7):688-95.
14. Liu H, et al. JMJD5 couples with CDK9 to release the paused RNA polymerase II. *Proceedings of the National Academy of Sciences of the United States of America.* 2020;117(33):19888-95.
